# Strategies for the Implementation of Chenodeoxycholic Acid in Natural Polymer-Based Hydrogels for Stable and Low-Light Effective Quasi-Solid Electrolytes for Aqueous Dye-Sensitized Solar Cells

**DOI:** 10.1021/acsaem.5c02940

**Published:** 2025-11-25

**Authors:** Lorenzo Casoli, Ana Yancy Segura Zarate, Elvira Maria Bauer, Matteo Bonomo, Simone Galliano, Claudia Barolo, Angelo Lembo, Lorenzo Gontrani, Marilena Carbone

**Affiliations:** † STARTNETICS—Department of Chemical Science and Technologies, 9318 University of Rome Tor Vergata, Via della Ricerca Scientifica 1, Rome 00133, Italy; ‡ Department of Chemistry, NIS Interdepartmental Centre and INSTM Reference Centre, University of Torino, Via Gioacchino Quarello 15/a, Torino 10135, Italy; § Escuela de Fisica, Instituto Tecnologico de Costa Rica, Cartago 159-7050, Costa Rica; ∥ Institute of Structure of Matter—Italian National Research Council (ISM–CNR), c/o Area della Ricerca di Roma1, Strada Provinciale 35d n. 9, Montelibretti 00010, Italy; ⊥ Italian National Research Council—Institute of Science, Technology and Sustainability for Ceramics (ISSMC−CNR), Via Granarolo 64, Faenza 48018, RA, Italy; # ICxT Interdepartmental Center, 9314 University of Turin, Lungo Dora Siena 100, Torino 10153, Italy

**Keywords:** hydrogels, natural polymer, quasi solid-state electrolyte, dye-sensitized solar cells, chenodeoxycholic acid, sustainability

## Abstract

The disclosing of the full potential of aqueous Dye-Sensitized Solar Cells (a-DSSCs) is tightly bonded to the implementation of more sustainable, stable, and efficient electrolytes which are able to perform both in outdoor and indoor environments. In this contribution, we report the formulation of biopolymer-based hydrogels (e.g., chitosan, carrageenan, and porcine gelatin) in conjunction with ZnO nanoparticles and their application as electrolytes in quasi-solid-state a-DSSC. The thorough characterization (XRD, IR, SEM) of the hydrogels reveals tunable morphology impacting their ability to stabilize a I-based redox mediator and chenodeoxycholic acid (CDCA) employed as an additive. Once implemented in a-DSSCs, all the biopolymer-based quasi-solid electrolytes perform well, delivering a photoconversion efficiency (PCE) approaching or even overcoming 1% (comparable to the one of a reference cell based on xanthan gum), showing an extremely high open circuit voltage (>700 mV). The addition of CDCA, alternatively during the gel formulation or washing phase, enables the porcine gelatin-derived systems to increase their efficiency of about 50% and carrageenan ones to ensure an extremely promising shelf life with any PCE decrease over more than 15 days. More importantly, the porcine gelatin-derived, CDCA-additivated electrolytes are able to properly perform also under indoor light (i.e., 1200 lx), showing PCE values approaching 3% and outperforming reference devices. Our results prove how a thoughtful selection of the polymer scaffold and a specifically designed preparation strategy are fundamental to improve the efficiency of sustainable quasi-solid aqueous electrolytes.

## Graphical abstract

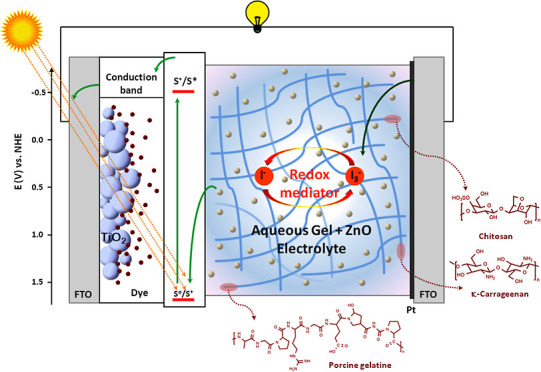

## Introduction

Hydrogels are hydrophilic polymer networks capable of retaining substantial amounts of water within their three-dimensional structure.^1^ The interplay between the liquid and the polymeric matrix and the possibility to host functional additives allows for the design of customized gels, thus having a tremendous impact on the wide applicability of these classes of materials.^2,3^ Recent efforts focused on the use of natural polymers (alginate, cellulose, chitosan, collagen, and gelatin among others) as jellying agents aim to improve the biocompatibility, biodegradability, and overall sustainability of the final hydrogels.^4^ Moreover, the possibility of chemically modifying the backbone of natural polymers paves the way for the customization of hydrogel properties to meet specific needs.^5^ The innate biological compatibility of a natural polymer-based hydrogel allows extensive use in the biomedical sector as drug delivery systems or in tissue engineering.^6^ For instance, chitosan-based hydrogels exhibit excellent antimicrobial properties,^7^ whereas alginate-based ones are widely used in cell encapsulation.^8^ Besides biomedical applications, biobased hydrogels have also made significant inroads into environmental and industrial sectors. In agriculture, they are used as soil conditioners to improve water retention and nutrient delivery, aiding in plant growth, especially in arid regions.^9^ Hydrogels have been also successfully exploited in water purification processes, playing the role of absorbents helping in the removal of heavy metals.^10^ More importantly, water-based hydrogels are garnering increasing attention as safe, sustainable, and cheap electrolytes in both energy harvesting (e.g., photovoltaic)^11–13^ and storage (e.g., batteries, capacitors)^14,15^ devices due to their ability to maintain a good ionic conductivity (related to the liquid phase) preventing leakages and ensuring mechanical stability and safety typically shown by (quasi-)­solid electrolytes. Moreover, the tunability of hydrogel properties through polymer composition, cross-linking density, and incorporation of functional additives allows for the design of tailored electrolytes.

In the field of material science, the use of inorganic nanoparticles (NPs) as jellying agents represents a dramatic advancement by combining the functionalities of NPs and the mass and charge transport features of the liquid phase: indeed, the use of metallic (e.g., Au, Ag) or metal oxide (e.g., SiO_2_, Fe_x_O_y_) NPs would dramatically impact the conductivity and the mechanical stability of the hydrogels. On the one hand, NPs could exploit their innate antimicrobial properties by being stabilized by the hydrogel network; on the other hand, iron oxide NPs could introduce magnetic responsiveness in the hydrogel.^16^ In the environmental chemistry realm, hydrogels added with TiO_2_ or ZnO have been proven to be effective in photodegradation of organic pollutants.^17^ More interestingly, the integration of inorganic NPs into biopolymer-based hydrogels not only broadens their application scope but also opens innovative avenues in developing smart, multifunctional materials by leveraging the biocompatibility and biodegradability of natural polymers while benefiting from the unique characteristics of nanoparticles.^1,18^

Within the photovoltaic field, hydrogels have shown significant promise as sustainable electrolytes in Dye-Sensitized Solar Cells (DSSCs),^19,20^ a class of devices that mimics natural photosynthesis, exploiting a dye (as catcher of the sunlight radiation) chemisorbed onto an inorganic semiconductor (e.g., TiO_2_) as the photoanode. In conventional DSSCs, the electronic circuit is closed by an electrolyte that regenerates the dye and is itself regenerated at the counter electrode. In these devices, the electrolyte plays a crucial role in terms of both efficiency, being responsible for the charge transport, and stability. As a matter of fact, traditional organic electrolytes, although performing, suffer from issues such as leakage and volatility, jeopardizing the long-term practicality of DSSCs.^21^ Aqueous hydrogels offer an innovative solution to these challenges. More interestingly, they have been proven to often outperform their liquid counterparts in stability.^22^ For example, some of us reported the use of xanthan gum as a jellying agent for quasi-solid DSSCs based on both I^–^/I_3_^–^ ^12^ or Co­(II)/Co­(III)^19^ couples as redox mediators, exceeding 2% and 4% as photovoltaic efficiency, respectively. In both cases, exceptionally high long-term stabilities up to a total time of 1200 h accompanied by even increased PCE efficiencies have been ascertained.

Furthermore, the incorporation of specific functional groups onto the polymeric matrix and/or nanoparticles into hydrogels can enhance their photoelectrochemical properties. Additionally, the mechanical flexibility and durability of hydrogels make them particularly suitable for flexible and wearable solar cell applications.^23^ The use of metal oxide nanoparticles as a gelling agent for acetonitrile to obtain quasi-solid organic electrolytes has been proposed by Chang et al.^24^ approaching or even overcoming the efficiency threshold of 4% by specifically tuning the amount of NPs to be dispersed into the solvent: 15% w/w of TiO_2_, 3% w/w of SiO_2_, or 35% w/w of ZnO to reach 3.74%, 3.63%, and 4.17%, respectively. Later on, Venkatesan et al.^25^ solidified a 3-methoxypropionitrile-iodide (MPN) electrolyte by addition of poly­(ethylene oxide)­(PEO)–poly­(methyl methacrylate)­(PMMA)–10% TiO_2_ NPs obtaining printable gel electrolytes suitable for QS-DSSC fabrication with up to 9.12% efficiency. More recently, the same research group also presented printable polymer electrolytes based on only PEO using Al_2_O_3_ and ZnO NPs. Application of the MPN-iodide-based gel electrolytes in DSSCs resulted in efficiencies of 8.73% and 7.59%, respectively.^26^ A step forward toward NPs/biopolymer quasi-solid hydrogels was made by Mary Johnson Leeda Rani et al.^27^ who proposed both Mn_3_O_4_ and CuS as additives of a Locust Gum-based hydrogel reaching a photoconversion efficiency close to 3.5%. However, one should note that the solvent is a 1:1 volumetric mixture of deionized water and organic solvent (ethylene carbonate and propylene carbonate), preventing us from classifying this electrolyte as fully aqueous and fully green. Quite recently, more sustainable quasi-solid-state aqueous electrolytes containing metal oxide nanofillers have been presented by Solikah et al. and Shafa et al.^28,29^ The authors proposed acetic acid-based chitosan/polyaniline/KI/I_2_ gel electrolytes reinforced by TiO_2_ or ZnO NPs as promising candidates for integration in DSSCs but the performance of the modified electrolytes in solar cell devices was not reported.

Very recently, we proposed a hybrid hydrogel, as electrolytes in quasi-solid aqueous DSSCs based on the biopolymer galactomannan and on Zn-derived NPs synthesized by a green and energy-saving route.^11^ The resulting hydrogel, once loaded with NaI and I_2_ as redox mediators and implemented in a complete device, showed similar photoelectrochemical properties of a reference quasi-solid device using a xanthan gum-based hydrogel, but showed much higher photovoltage (i.e., 700 vs 550 mV). Such a value represents the state of the art of these types of devices. Taking the cue from these results, we decided to extend our research by employing other biopolymer/Zn-based NP combinations as gelling agents and testing them as green and effective electrolytes in quasi-solid aqueous DSSCs. Aiming at employing green, cheap, and easily available biopolymers, we chose to probe the efficacy of porcine gelatin, carrageenan, and chitosan in combination with ZnO NPs. These gels were selected based on their distinct structures, which may lead to different interactions with the ZnO NPs and the redox mediator in the DSSCs, as well as their diverse natural origins.

Carrageenan is a natural sulfated polysaccharide extracted from red seaweed species and is commonly used as a food additive, primarily as a thickening, gelling, and stabilizing agent.^30^ With respect to its application in DSSCs, Bantam and Chamaco explored the use of *k*-carrageenan as a gelling agent, obtaining moderate efficiency (i.e., <0.3%) only if ACN is used as a cosolvent.^31^ Quite interestingly, the same authors further engineered their gel by implementing inorganic nanoparticles (e.g., TiO_2_ or Fe_2_O_3_) approaching a PCE of 0.8%.^32^ Chitosan is a versatile and ecofriendly biopolymer, belonging to the family of aminopolysaccharides. It is derived from chitin, a natural substance found in the exoskeletons of arthropods such as shrimp, crabs, and lobsters, and is widely employed in various industries due to its gel-forming ability and biodegradability.^27^ Albeit largely used as a gelling agent in organic solvent-based electrolyte (approaching 2% with ethylene carbonate as a solvent and TiO_2_ nanoparticles as cogelling agents^33^), chitosan in 100% aqueous redox mediator led to a very poor efficiency (0.06%).^34^ Porcine gel is obtained by extracting and hydrolyzing collagen from pig skin, making it primarily composed of polyamino acids rich in glycine, proline, and hydroxyproline, which contribute to its gel-forming capabilities^35^ and differently from chitosan and κ-carrageenan has never been exploited as a gelling agent in aqueous DSSCs, as far as we are aware.

We carried out our investigations by fully characterizing the ZnO gels in their native, dried, or freeze-dried forms using XRD, SEM, and FT-IR. Subsequently, well-performing quasi-solid and semitransparent aqueous Dye-Sensitized Solar Cells (DSSCs) were assembled using an iodine-based redox mediator, achieving a photoconversion efficiency (PCE) in the 1% range. The further addition of chenodeoxycholic acid (CDCA) to the porcine-gel-based electrolyte, never explored in such systems, resulted in variations of the gel morphology, depending on the timing of addition. In the best case, this led to a PCE approaching 1.6%.

Our results demonstrate that a thoughtful selection of the polymer scaffold and a specifically designed preparation strategy are crucial for improving the efficiency of sustainable quasi-solid aqueous electrolytes.

## Experimental Section

### Materials

Zinc nitrate (Zn­(NO_3_)_2_·6H_2_O, CAS 10196-18-6), sodium hydroxide (NaOH, CAS 1310-73-2), sodium iodide (NaI, CAS 7681-82-5), iodine (I_2_, CAS 7553-56-2), chenodeoxycholic acid (CDCA, CAS 474-25-9), absolute ethanol (EtOH, CAS 64-17-5), acetone (CAS 67-64-1), tert-butanol (*t*-BuOH, CAS 75-65-0), isopropanol (2-prop, CAS 67-63-0), fluorine-doped tin oxide-coated glass slide (FTO glass, surface resistivity ∼7 Ω sq^–1^), titanium tetrachloride (TiCl_4_, CAS 7550-45-0), chloroplatinic acid (H_2_PtCl_6_, CAS 16941-12-1), and 2-Cyano-3-[4-[4-(2,2-diphenylethenyl)­phenyl]-1,2,3,3a,4,8b-hexahydrocyclopent­[b]­indol-7-yl]-2-propenoic acid (D131 dye) were purchased from Sigma-Aldrich. Deionized water (DI-H_2_O, 18 MΩ cm at 25 °C) was obtained with a Direct-Q 3 UV Water Purification System (Millipore). TiO_2_ paste (DSL 18 NR-T) was purchased from Dyesol. Porcine gelatin (CAS 9000-70-8) was obtained from Sigma or Fluka, chitosan (CAS 9012-76-4) from Acros Organics and Sigma-Aldrich, κ-carrageenan (CAS 9000-07-1) was acquired from Sigma-Aldrich, and *N*-acetyl cysteine (NAC, CAS 616-91-1) from Alfa-Aesar. Glacial acetic acid (CAS 64-19-7) was purchased from Carlo Erba.

### ZnO-NP Gel Syntheses

The synthesis of the gels is a multistep procedure, comprising the preparation of the starting polymer/water solutions, zinc salt addition, precipitation reaction, and solution centrifugation leading to gel formation, and it is part of a green synthetic approach of ZnO and ZnO-based materials.^36,37^ A schematic presentation of all hydrogel preparations and subsequent treatments (drying, addition of I-based redox mediator NaI/I_2_ and CDCA additive treatments) is given in Scheme 1, while a complete list of all samples can be found in Table 1.

**Scheme 1:**
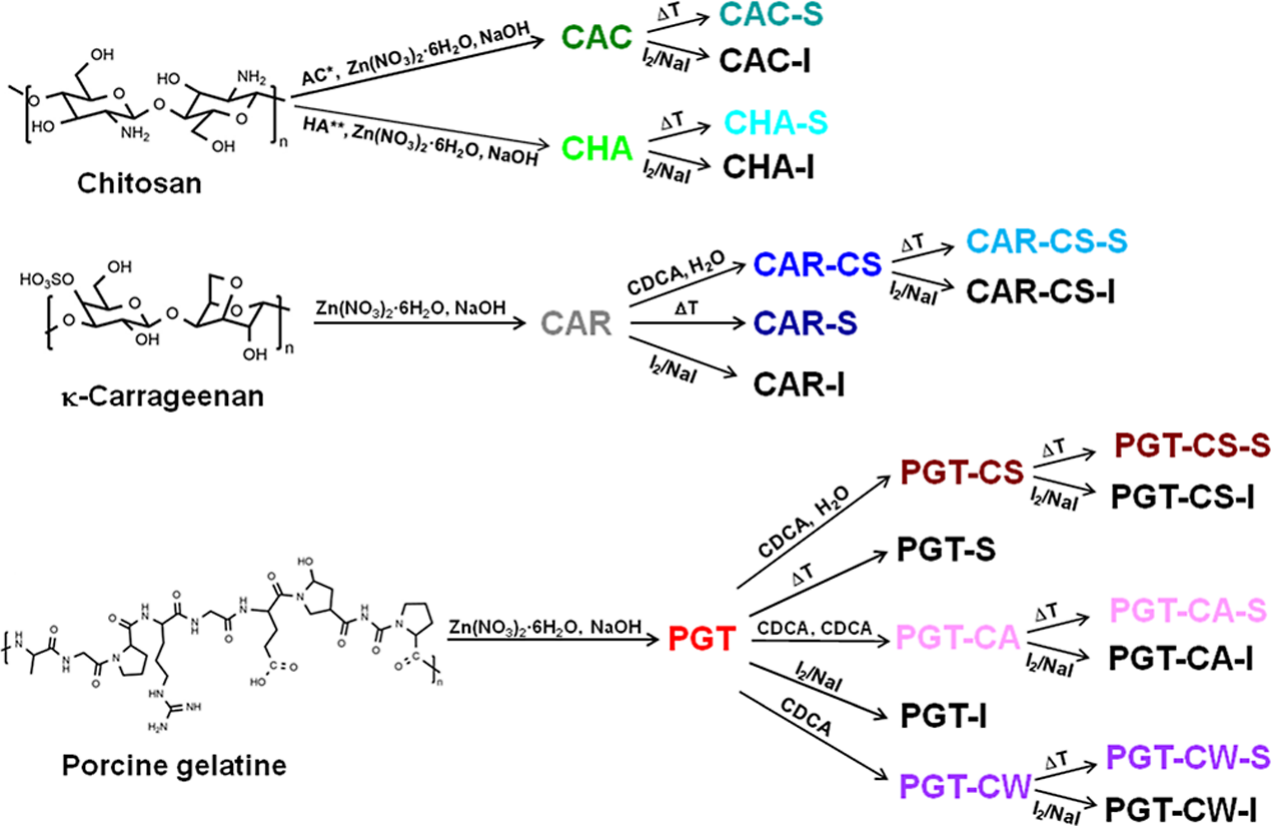
Synthesis of Chitosan-, Carrageenan-, and Porcine Gelatin-Derived ZnO Hydrogels. AC* = *N*-Acetylcysteine, HA** = Acetic Acid

**Table 1:** Correspondence between Labels and Samples. The Label **-S** after the Name Stands for Solid, **-I** for Iodide Loaded

label	biopolymer	additional treatment	dried Sample	iodide loaded
CAC	Chitosan/N-Acetylcysteine	-	CAC-S	CAC-I
CHA	Chitosan/Acetic acid	-	CHA-S	CHA-I
CAR	Carrageenan	-	CAR-S	CAR-I
CAR-CS	Carrageenan and CDCA-saturated DI water 1:1 in volume	washed with DI water	CAR-CS-S	CAR-CS-I
PGT	Porcine Gelatin		PGT-S	PGT-I
PGT-CS	PGT and CDCA-saturated DI water 1:1 in volume	washed with DI water	PGT-CS-S	PGT-CS-I
PGT-CA	PGT and CDCA-saturated DI water 1:1 in volume	washed with CDCA-saturated DI water	PGT-CA-S	PGT-CA-I
PGT-CW	Porcine Gelatin	washed with CDCA-saturated DI water	PGT-CW-S	PGT-CW-I

The starting solutions of carrageenan and porcine gel were prepared by dissolving them in 100 mL of deionized water in a 1% w/v proportion, followed by stirring at 60 °C for 24 h in an oil bath. As for chitosan, 100 mL of a 1% w/v solution was added either with 0.489 mg of solid *N*-Acetyl Cysteine (NAC) or with 1 mL of acetic acid to aid its solubilization. Samples corresponding to the latter preparation procedure are indicated as **AC** or **HA**, respectively.

After 24 h, Zn­(NO_3_)_2_•6H_2_O was added to the starting solutions at 2% w/v. The resulting solution was kept for 2 h at 60 °C in an oil bath under vigorous stirring. Finally, 100 mL of NaOH 0.1 M was added dropwise and left stirring overnight at 60 °C.

ZnO formed according to the following chemical reactions:

$${Zn\left({NO}_{3}\right)}_{2}+2NaOH\to {Zn\left(OH\right)}_{2\left(s\right)}+{2NaNO}_{3\left(aq\right)}\to {ZnO}_{\left(s\right)}+H_{2}O+{2NaNO}_{3\left(aq\right)}$$

Once the aging was complete, the mixture was centrifuged three times at 3500 rpm, 25 °C for 10 min, and rinsed with deionized water after each centrifugation. The biopolymer–nanocomposite gel formed during the centrifugation step as a deposit at the bottom of the vial (samples **CAC**, **CHA**, **CAR**, and **PGT**). For characterization purposes, a portion of the nanocomposites was collected and dried in an oven at 40 °C for 24 h. All dried samples examined in this work carry the extension **S** as, for example, sample **CAC-S**. Another aliquot underwent a freeze-drying cycle for 5 h at −61.5 °C and 330 Torr. Apart from the different dissolution time for porcine gelatin, all other conditions, i.e., zinc nitrate hexahydrate addition, NaOH addition, and reaction time, are unchanged.

Coming to CDCA-modified gels, the latter was introduced by using different approaches: (i) a saturated CDCA solution was mixed (1:1 v/v) with the ZnO NP–gelatin solution and centrifuged (3500 rpm, 10 min at 25 °C), obtaining a wet gel phase which is washed with deionized water for three times (samples **-CS**); (ii) at a variance with this procedure, saturated CDCA aqueous solution was also used in the washing phase (samples **-CA**); (iii) as a last attempt, the CDCA was introduced only after the first centrifugation step, i.e., using it as the washing solution (samples **-CW**).

### ZnO-NP Gel Characterizations

X-ray Diffraction (XRD) was carried out using an X’Pert pro X-ray diffractometer by Philips, operating with CuK-Alpha radiation on the dried samples. Infrared spectra were recorded both on gels and on dried samples with a Shimadzu Prestige-21 FT-IR instrument, equipped with an attenuated total reflectance (ATR) diamond crystal (Specac Golden Gate), in the 400–4000 cm^–1^ range, with a resolution of 4 cm^–1^. The morphology of the synthesized samples, both as gel, upon freeze-drying cycles, and as dried powder was determined by FE-SEM, Field Emission Scanning Electron Microscope AURIGA, Carl Zeiss SMT, Oberkochen (Germany) operating at 7 kV, after deposition on a silicon wafer, used as a sample holder.

### Device Assembly

Devices were assembled as reported in our recent paper.^11^ Very briefly, a transparent paste TiO_2_ was deposited by the screen-printing technique on the conductive side of a FTO glass, leading to an ∼6 μm-thick electrode (0.5 cm × 0.5 cm active area), followed by a sintering step at 450 °C for 30 min. The so-obtained photoanodes were dipped into a solution of TiCl_4_ (40 mM in deionized water, DI-H_2_O) for 30 min at 70 °C, rinsed with DI-H_2_O and EtOH, and further sintered at 450 °C for 30 min. The sensitization occurs by dipping the electrode in a dye solution (D131, Figure SI1 (0.5 mM)) and CDCA, as coadsorbents (ratio 1:34 dye to CDCA),^12^ in a mixture of ACN/*t*-BuOH (1:1) for 4 h at RT. Counter electrodes were obtained by drop-casting 40 μL of H_2_PtCl_6_ (5.0 mM solution in isopropanol) on the conductive side of a FTO glass and then sintered at 450 °C for 30 min.

The I-based redox mediators were prepared by adding 73.6 mg of NaI and 7.6 mg of iodine for every mL of the gel, leading to a final concentration in the hydrogel as high as 0.5 and 0.03 M for iodide and iodine, respectively. After the addition, the gels were vortexed at high speed (>3000 rpm) for at least 10 min, leading to the homogenization of the mixtures that turned dark orange at the end of the process. All aqueous gel electrolytes were stored in dark conditions at room temperature, and corresponding samples are indicated with −**I** extensions.

Aqueous gel electrolyte (∼2.5 mg) was then cast on the photoanode with a spatula; the photoanode and counter electrode were then glued together with a Surlyn© thermoplastic film (internal square area = 0.6 × 0.6 cm^2^) by hot pressing at 105 °C for 20 s leading to a final interelectrode distance of around 50 μm.

### Photovoltaic Characterization

Current density–voltage (J–V) curves under 1 sun light intensity (0.1 W/cm^2^, AM 1.5G) were recorded with a solar simulator (ABET Technologies Inc., model 10500, Milford USA) equipped with a 150 W xenon arc lamp and connected to a digital source meter unit (SMU 2420 Keithley, Tektronix, USA). Before each measurement, the source was calibrated with a silicon reference solar cell (irradiation sensor, Spektron 210, 74.63 mV). For indoor-light measurements, J–V curves were recorded under 1200 lx (0.414 mW/cm^2^) provided from a cool white LED (4100 K) driven by a programmable current source. The power of the white LED was measured with a radiometer (HD 2102.2, Delta Ohm, Italy) coupled with an irradiance probe (LP 471 RAD, Delta Ohm, Italy).

The transmittance of the complete cells was recorded with a UV–visible double-beam spectrophotometer (Varian Cary 300 Bio, Series II, Agilent Technologies, Australia) in the wavelength range of 300–800 nm, using air as a reference.

## Results and Discussion

### Gel Characterization

The prepared gels were dried at 40 °C for 24 h in order to minimize the amount of absorbed water and were characterized by XRD, with the aim of identifying the phases present in the materials and of assessing their degree of crystallinity. In order to point out the presence of crystalline peaks in the scattering profile, the baseline originated from the amorphous contribution due to the sample holder and to the sol phase of the hydrogel was subtracted from raw scattering data, using either the pattern measured for the plain gel (i.e., containing no nanoparticles) or a convex hull distribution.^38^ Among all the cases examined, only in the spectra of PGT containing samples (**PGT**, **PGT-CA**, and **PGT-CS**) as well as in CAR samples (Figure 1a–c and d, respectively), evident sharp crystal peaks could be identified in the scattering profiles, whereas in the two remaining patterns (**CHA** and **CAC**, Figure 1e and 1f, respectively), only the featureless diffuse scattering from the gel is visible, signaling that the embedded nanocrystallites are either totally absent or present in negligible concentration, therefore causing the signal coming from the hydrogel to completely overwhelm their scattering features. The pruned diffraction profiles of **PGT**, **PGT-CA**, **PGT-CS**, and **CAR** were analyzed with GSAS-II software^39^ according to Rietveld fitting procedures, that refine peak intensities and crystal cell edges of the phase. As putative zinc compounds hypothesized to be present, hexagonal zinc oxide (Wurtzite-like zincite mineral, space group *P*6_3_mc) and orthorhombic ε-Zn­(OH)_2_ (space group *P*2_1_2_1_2_1_, the most stable phase of zinc hydroxide, found in Wulfingite mineral)^40^ were employed in the Rietveld fit, in line with some previous reports by some of us.^37^ The necessary crystallographic data were collected as CIF files from the American Mineralogist Crystal Structure Database^41^ or directly from ref 32. The theoretical powder patterns were calculated by convoluting a series of pseudo-Voigt functions, modified for asymmetry, each centered on the crystal peak.^42^ Concerning PGT containing specimens, the analysis indicates that in both cases, ZnO nanocrystallites are present in the dried gel, as evidenced by the high intensity “triad” of hexagonal ZnO reflections, occurring at 32.0°, 34.5°, and 36.3°, and corresponding to the reflections from 100, 002, and 101 planes, respectively, together with smaller but clear peaks falling at higher angles. The intensity profile of the triad (medium–low–high) is very similar to the progression observed in crystalline ZnO or in samples composed of spherical nanoparticles,^43^ indicating no preferential directions and isotropic crystal growing. The fitted crystal data for the three samples are quite similar and range between 3.248 and 3.254 Å (*a* axis), 5.212–5.213 Å (*c* axis), V = 47.634–47.669 Å^3^ (cell volume), 5.657–5.670 g cm^–3^ (density), atom parameters (fractional coordinates): Zn *x* = 0.333, *y* = 0.667, *z* = −0.023–0.025; O *x* = 0.3337, *y* = 0.667; *z* = 0.365–0.370. The average crystallite size, calculated by the fitting software according to the Scherrer equation relating the latter dimension to the radiation wavelength λ, to the scattering angle θ, and to Full Width at Maximum Height (FWMH) of all the fitted peaks, is 73.6 nm for **PGT-CA** and 51.2 nm for **PGT-CS**. However, such values should be considered with care as crude estimates, since the crystallites were soaked into the complex hydrogel matrix before the drying, and the matrix might have caused a large swelling of the nanoparticles. Besides ZnO reflections, other fainter through definite peaklets/shoulders can be distinguished, in particular the features noticeable at 33.0° and 58.5°. These peaks can be traced back to the reflections of ε-Zn­(OH)_2_ with good confidence, hinting at the presence of an oxide–hydroxide mixture in the dried samples. The Rietveld fit, using the mole fractions of oxide and hydroxide as fit variables, yielded a putative composition 98.5% ZnO–1.5% Zn­(OH)_2_ for **PGT**, 98% ZnO and 2% Zn­(OH)_2_ for **PGT-CA**, and 90% oxide–10% hydroxide for **PGT-CS**, suggesting a slightly larger hydroxide content in the samples that were rinsed with DI water after CDCA addition. Yet, it should be considered that the drying process prior to XRD measurements might also induce a conversion of Zn­(OH)_2_ into ZnO and therefore an overestimation of the oxide/hydroxide ratio with respect to the one predicted by the Rietveld analysis. In Figure 1d, the XRD pattern of the **CAR** sample is shown. Despite the general agreement with an XRD profile generated by a system containing ZnO nanoparticles, as already discussed for PGT samples, some noteworthy features should be underlined. The most striking one is the different progression of peak intensities of the hexagonal lattice signals (ZnO triad): remarkably, in **CAR**, the peaks follow the order low–medium–high, that is different from that owned by bulk ZnO or isotropic nanoparticles,^43^ pointing out that the crystallites show some degree of alignment preference along 002 crystallographic directions, given the large intensity gain of the central peak (002). This feature may be due to a likely sizable templating effect of the polymer matrix (the polysaccharide carrageenan), that has been shown to often adopt helical or double helical structures,^44^ an effect that appears to be maximized during the gelation process, when a coil to helix transition is observed, followed by the final restructuring of helices into three-dimensional extended networks;^45–47^ additionally, cations often promote gelation phenomena.^48^

**Figure 1:**
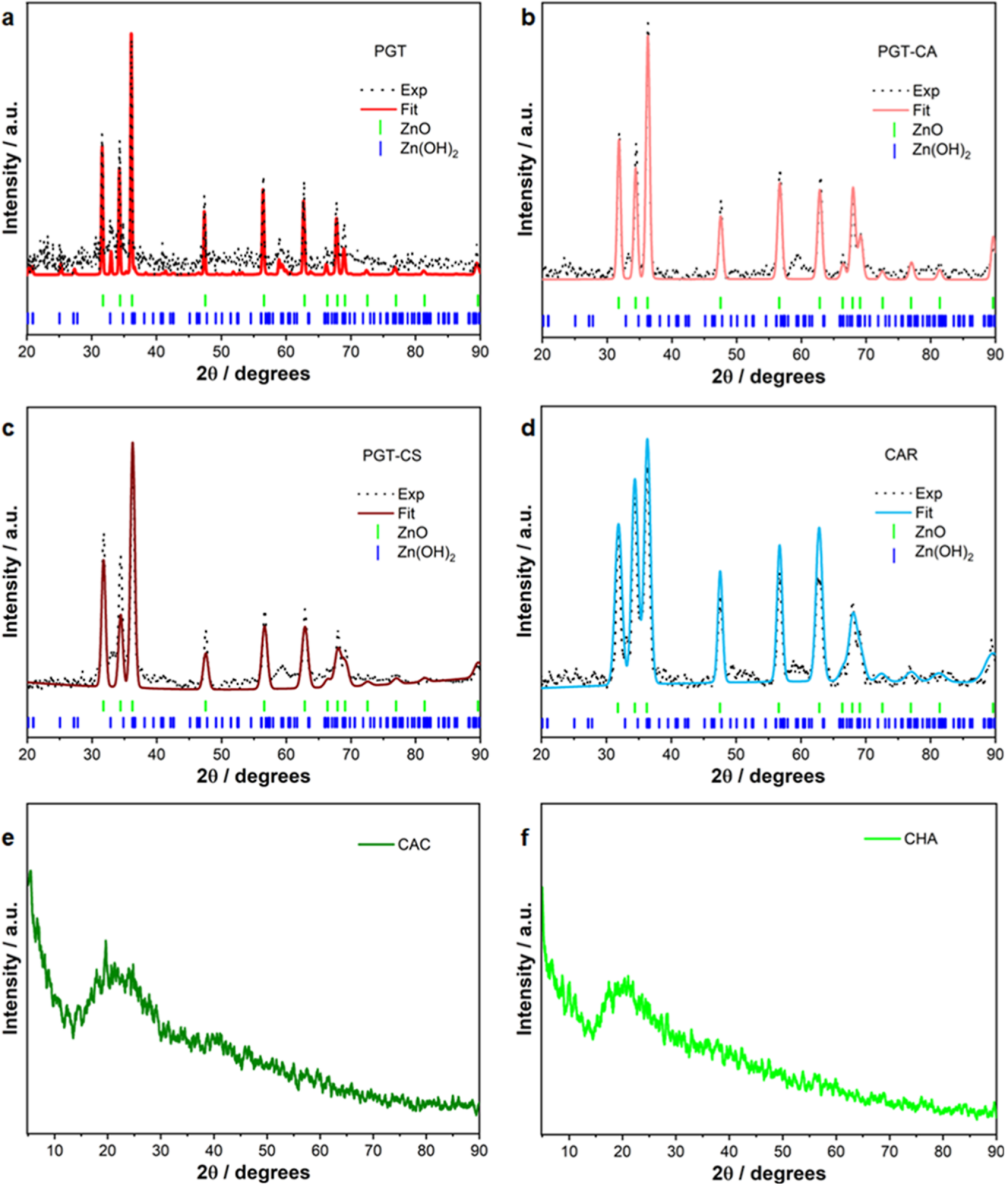
XRD patterns of (a) **PGT**, (b) **PGT-CA**, (c) **PGT-CS**, (d) **CAR**, (e) **CAC**, and (f) **CHA**. The vertical lines correspond to ZnO (green) and Zn­(OH)_2_ (blue). In panel a–d, black dots: experimental data; continuous line: Rietveld fit. In panels e and f, dark-green line: **CAC** pattern, light-green line: **CHA** pattern.

In order to properly fit the diffraction pattern, the extent of preferred orientation along the 002 direction was added as a parameter to the Rietveld fit, within the framework of the March–Dollase theory.^49^ In particular, the fitting reported a March–Dollase ratio of 0.86, representing the crystallite fraction with reciprocal lattice vectors normal to the 002 direction. The other fitting parameters are in line with **PGT-CA** and **PGT-CS**: *a* = 3.245 Å, *c* = 5.215 Å, *V* = 47.571 Å^3^, *d* = 5.681 g/cm^3^; the Scherrer analysis reports an average crystallite dimension in the micrometric range (around 10 μm). Regarding the additional peaks ascribable to Zn­(OH)_2_ (at 33° and 58.5°), only the latter one is sizable in this case; overall, a similar oxide:hydroxide ratio can be predicted for the **CAR** sample as well. It should be noticed at the end of this session that the final CDCA washing (CW), followed by drying, led to samples whose XRD profiles are substantially equivalent to those obtained for the same compound with other settings and are omitted for the sake of simplicity of the panel arrangement in Figure 1.

In Figure 2a, *N*-acetyl cysteine-chitosan-based ZnO hydrogel (**CAC**) and chitosan-based gel (**CHA**) and derived solids (**CAC-S** and **CHA-S**) are compared to the starting solid reagents chitosan and *N*-acetyl cysteine. In this regards, *N*-acetyl cysteine shows characteristic vibrations related to N–H stretching of the CONH carboxamide group (3370 cm^–1^), S–H stretching (2546 cm^–1^), C=O stretching of carboxylic ions (1713 cm^–1^), and C=O stretching of carboxamide (1530 cm^–1^).^50^ Broad overlapping O–H and N–H stretching vibrations centered around 3290 cm^–1^, C–H stretching modes (2918 and 2872 cm^–1^), overlapping C=O stretching of residual acetyl groups and NH_2_ stretching (1658–1570 cm^–1^), asymmetrical CH_2_ bending (1418–1377 cm^–1^), and C–O bridge stretching of glucosamine residues (1030 cm^–1^) are instead typical of the chitosan reagent used in this study.^51^ The absence of signals above 1700 cm^–1^ in the latter indicates a lack of carboxyl groups. Upon addition of acetic acid, the free amine groups in chitosan are likely converted into NH_3_^+^ forming a soluble cationic polyelectrolyte. Interestingly, both gels and solids showed similar vibration patterns which could be related to completely deacetylated chitosan, i.e., the vibration around 1650 cm^–1^ disappears. The water content in **CAC** and **CHA** is very high as ascertained by intense and broad OH stretching and bending modes centered around 3290 cm^–1^ and 1620 cm^–1^, respectively. Transmittance decreases below 500 cm^–1^ due to formation of Zn–O bonds that have not been detected in the solid samples **CAC-S** and **CHA-S**, while in both gels (**CAC** and **CHA**), an evident peak around 524 cm^–1^ generally related to formation of Zn–N bonds can be observed.^52^ The latter signal nearly disappears upon dehydration, indicating that its intensity strongly depends on the water content. Interestingly, Perelshtein et al. observed that sonochemical treatment of zinc acetate in the presence of chitosan at pH = 8 results in formation of zinc oxide nanoparticles smaller than 2 nm which are not detectable by XRD analysis.^53^ FT-IR spectra of their isolated composites also showed several vibrational peaks below 600 cm^–1^. Furthermore, zinc–chitosan complexes have been obtained by Wang et al. from zinc sulfate at pH = 7 whose FT-IR spectra between 1700 and 1000 cm^–1^ closely resemble our experiments performed at pH = 6.^52^ Therefore, based on the latter observations, also in our chitosan samples, formation of zinc oxide composites or zinc–chitosan complexes cannot be excluded. The similarities among all chitosan-based compounds are most probably due to reprecipitation of chitosan upon addition of NaOH solution until pH = 6. Indeed, the p*K*_a_ value of the primary amino groups of chitosan (6.3) is close to the final pH of the reaction media, providing the right conditions for partial precipitation of chitosan even after initial acetate salt formation.^51^ The latter assumption is in line with the previously discussed XRD results of the chitosan-based compounds.

**Figure 2:**
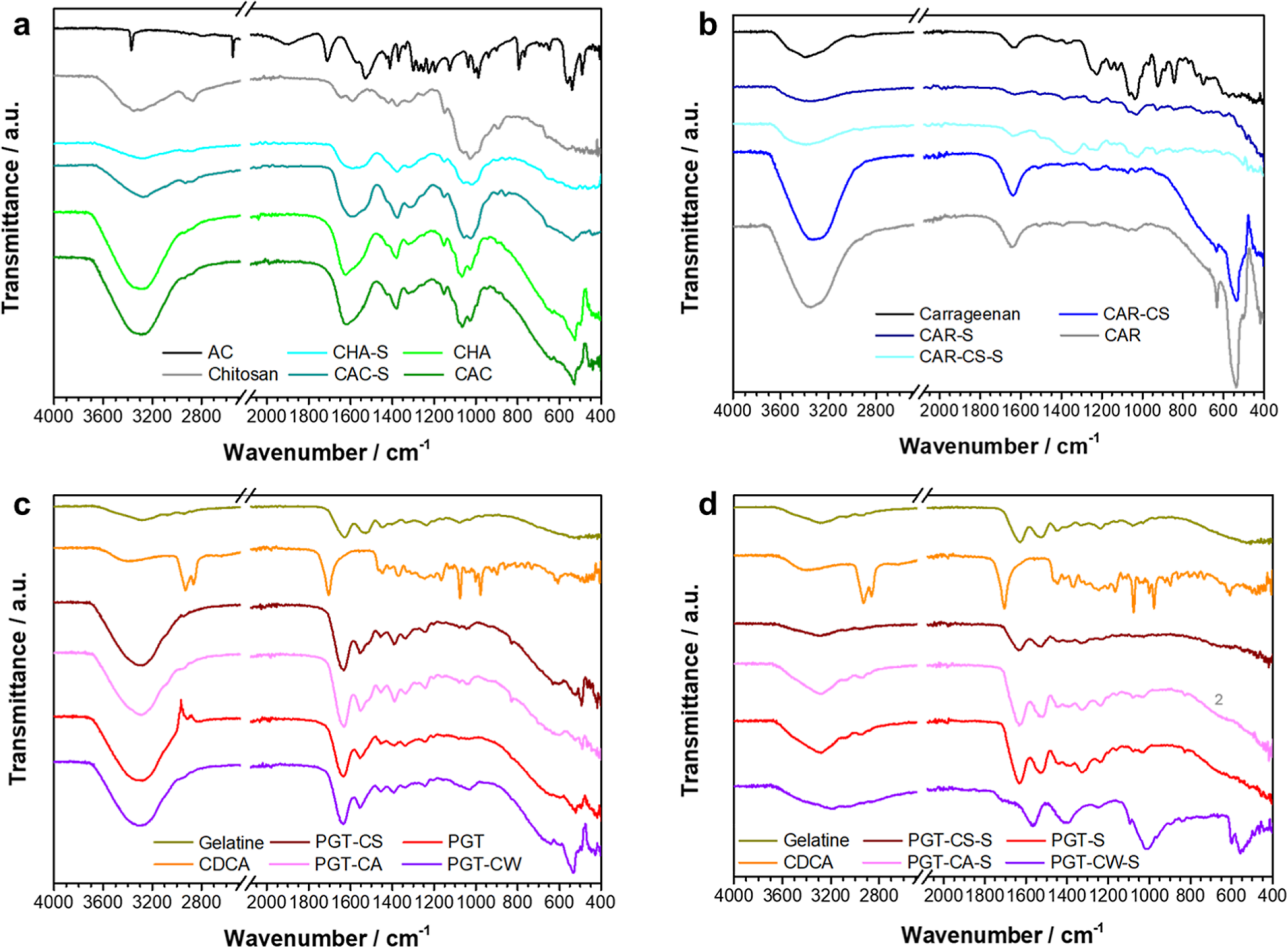
FT-IR spectra of the different gels employed throughout the paper. Data are clustered for an easier comparison: (a) chitosan-based gels and dried samples, (b) carrageenan-based gels and dried samples, (c) gelatin-based gels, and (d) gelatin-based dried samples.

Also, the carrageenan-based gels (Figure 2b) **CAR** and **CAR-CS** are dominated by evident signals due to the high water content such as OH stretching around 3330 cm^–1^, H–O–H bending at 1637 cm^–1^, and the humidity-dependent signals at 634 and 536 cm^–1^. Typical vibrations of the carrageenan biopolymer^54^ related to OH stretching centered around 3370 cm^–1^, C–H asymmetric and symmetric stretching at 2964 and 2899 cm^–1^, respectively, S–O or C–O sulfate ester stretching at 1225 cm^–1^ and 1155 cm^–1^, respectively, sulfate ester bending at 845 and 800 cm^–1^, C–O stretching of primary alcohol at 1065 cm^–1^, or C–O–C stretching of 3,6-anhydro-d-galactose at 924 cm^–1^ are barely visible in the gels or solids **CAR-S** and **CAR-CS-S**. A deeper inspection of the dried sample **CAR-S** shows a broad peak below 600 cm^–1^ together with weak vibrations between 486 and 416 cm^–1^ characteristic of Zn–O bonds. In **CAR-CS-S**, several weak peaks can be spotted below 500 cm^–1^, but in this case, the typical broad absorption of ZnO below 600 cm^–1^ is missing. Interestingly, in the latter sample, broad peaks in the spectral range between 1350 and 1500 cm^–1^ can be observed. Here, chenodeoxycholic acid has been added during the first washing step whose carboxylic group can interact with zinc ions resulting in asymmetric and symmetric COO^–^ stretching vibrations located around 1550 and 1410 cm^–1^, respectively.^55^

In Figure 2c and 2d, porcine gelatin-derived samples are reported together with powdered porcine gelatin and chenodeoxycholic acid (CDCA) used for different washing procedures. Also, in the latter gels (Figure 2c), intense and broad OH stretching (∼3306 cm^–1^) and bending vibrations (∼1630 cm^–1^), slightly shifted toward lower wavenumbers in respect to the chitosan- or carrageenan-derived gels, are present. Strong signals around 634–535 cm^–1^ are only detectable in sample **PGT-CW**, i.e., the specimen simply washed with saturated **CDCA** solution after centrifugation, which seems to contain more water than the other gelatin gels. In addition, the spectral features of **PGT-CW** nearly coincide with those registered for **PGT** between 1700 and 700 cm^–1^, indicating that simple washing with a saturated CDCA solution seems not to provide any sign of the chenodeoxycholic acid additive but recalls only vibration broadening. This is somehow expected, considering that the solubility of CDCA in water is lower than 1 mg/mL. On the other side, **PGT-CS** and **PGT-CA** show weak shoulders above 1500 cm^–1^ and enhanced vibration peaks between 1450 and 1390 cm^–1^ which may be related to the interaction of the carboxylic group of **CDCA** with zinc ions similar to what has been detected in gel **CAR-CS**. Most importantly, all samples are characterized by several evident signals below 500 cm^–1^ typical for Zn–O bonds. In the dried gels (**PGT-S**, **PGT-CS-S**, and **PGT-CA-S**), correspondence with pork gelatin characteristics is even more pronounced especially for samples **PGT-S** and **PGT-CA-S**. C=O stretching (amide I, 1624 cm^–1^), N–H bending and C–N stretching (amide II, 1527 cm^–1^), aliphatic C–H bending (1446, 1410, and 1333 cm^–1^), C–N bond stretching (amide III, 1240 cm^–1^), together with C–H, O–H, and N–H stretching modes (2881 cm^–1^, 2945 cm^–1^, ∼3280 cm^–1^, respectively) are assigned to gelatin.^56^ Formation of ZnO seems not to influence the spectral features of gelatin in this region suggesting no significant interaction between the functional groups of the biopolymer and zinc ions.^57^ **PGT-S** and **PGT-CA-S** showed both a remarkable decrease in transmittance below 600 cm^–1^ and several vibrations below 500 cm^–1^ generally associated with ZnO nanoparticles.

The morphology of the gel is expected to dramatically impact the final (photo) electrochemical properties. In Figure 3a–h, the most representative SEM images of the purposely freeze-dried samples are reported. In general, the textures and the morphologies are rather different for the 3 main sets of samples (carrageenan, chitosan, and pork gelatin derived). The most striking differences can be appreciated by comparing Figure 3a, c, and e among them, representing the samples **CAR**, **CHA**, and **PGT**, respectively. The **CAR** sample is characterized by ribbons and ripples (highlighted by orange rings) interspersed with segregated islands, that appear as composed of nanoparticles with an average diameter of 40 ± 4 nm, as can be better seen at higher magnification in Figure SI2a of the Supporting Information. **CHA** (as well as **CAC**, not shown) has a very compact texture that creates complete blocks of polymer extending over micrometers in all directions. In Figure 3c, a rectangular section of the polymer block is clearly visible. The magnification of any region of the block polymer indicates the presence of closely contiguous nanoparticles with an average diameter of 16 ± 3 nm (Figure 3d). Due to the compact nature of the blocks, the homogeneity, and regularity of the overall structure, which is actually made of repetitively contiguous nanoparticles leaving no space for areas rich in polymer as for the **CAR** sample, it can be hypothesized that the observed particles are composed of ZnO cores coated by chitosan, with the overall observed diameter. This assumption is fully compatible with the IR spectral interpretation. The image of **PGT** in Figure 3e shows a rough surface with holes or channels ranging in diameter from 2 to 4 mm. Also, in this case, the morphology is fairly regular throughout the sample.

**Figure 3:**
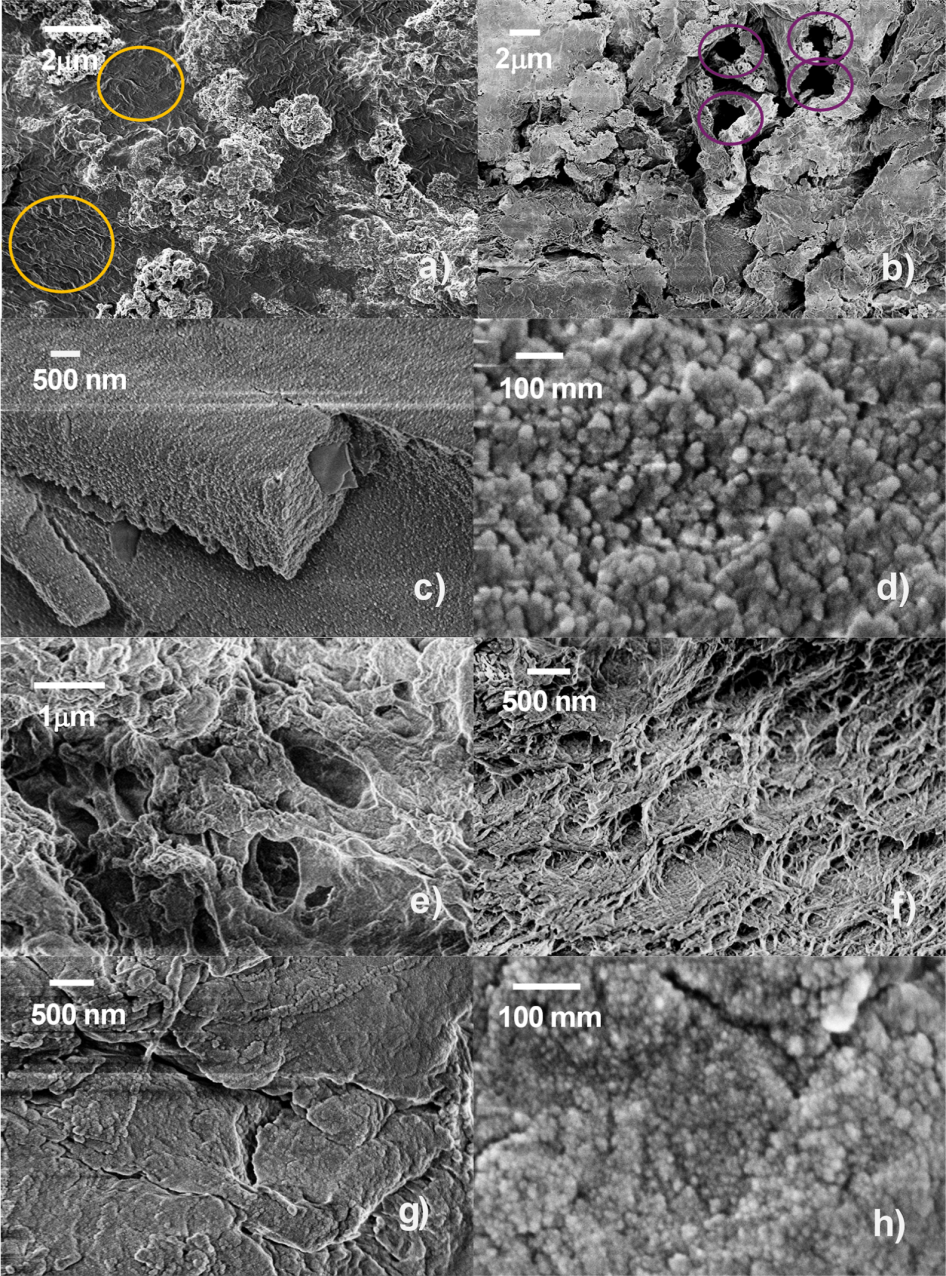
SEM images of the most interesting gels discussed throughout the work: (a) **CAR**, highlighted with orange rings to indicate areas with ribbons and ripples; (b) **CAR-CS**, i.e., CAR treated with CDCA during gel formulation, with purple rings highlighting hollow channels; (c) **CHA**; (d) **CHA** at higher magnification (300 kX); (e) **PGT** pristine; (f) **PGT-CW** (PGT treated with CDCA during the washing phase); (g) **PGT-CS** (PGT treated with CDCA during gel formulation); (h) higher magnification (300 kX) of the PGT sample shown in panel (e) to highlight the nanometric texture of the PGT-based samples.

The addition of CDCA to **CAR** and **PGT** produces opposite effects on the two samples. When added to **CAR**, CDCA leads to a disruption in the structure, generating holes and channels, that were not present in the pristine sample (Figure 3b, highlighted by purple circles), which could be considered as a positive feature for the loading of the I-based redox mediator and for the ionic diffusion of the active species. On the other hand, CDCA has a compacting effect, instead, on **PGT** extent of which depends on the mode of the addition: it induces a substantial channel reduction and the formation of a regular rugosity pattern, when added in the rinsing phase (Figure 3f, –**PGT-CW**); it causes compacting of the whole texture, when added to the reaction mixture during the gel preparation phase (Figure 3g, **PGT-CS**). Further rinsing with a CDCA solution in **PGT-CA** does not induce further compacting (Figure SI2b of the Supporting Information). Albeit a more compact gel could lead to ion-diffusion limitation, it could positively impact on the effective and long-lasting hosting of the I-based redox mediator. The different behavior of CAR and PGT with respect to CDCA addition can be likely related to the composition of the gels, i.e., a sugar (CAR) or a protein and lipid mixture (PGT) and the capability of CDCA to form amphiphilic micelles,^58^ that can create an intermingling network in the presence of lipids.

The effect is larger if CDCA is added from the start but still significant when used in the rinsing phase. Finally, a large magnification of **PGT** images indicates the presence of a homogeneous texture at the nanometric level. This is reported in Figure 3h for **PGT** as an example, but it is common to all **PGT** samples.

### Photovoltaic Characterization of Quasi-Solid Aqueous DSSCs

Once the characterization of both the gels (in their pristine and dried fashion) had been completed, we proceeded to incorporate both I_2_ and NaI to obtain the electrolyte for the quasi-solid aqueous Dye-Sensitized Solar Cells. However, before the implementation of the electrolytes into the complete devices, we checked the effect of the addition of the redox mediator onto the stability of the different gels. Indeed, hydrogels are metastable and the addition of a salt (NaI) or a coordinating neutral species (I_2_) causes the modification of the intermolecular interaction and phase separation in the worst cases.^1^ On the one hand, **CAR** and **PGT** prove themselves to be able to properly host the redox couple, fully solubilizing both I_2_ and NaI and giving rise to a dark-orange gel; on the other hand, both **CAC** and **CHA** show a peculiar behavior. Both seem to be able to solubilize the redox couple under vigorous stirring, yet once the stirring is switched off, both the gels behave as metastable systems: after the addition of the redox mediator, a partial discoloration of the gel could be observed but without any phase segregation (see Figure SI3), thus suggesting a massive evaporation of the Iodine or its partial reduction to Iodide. This could be tentatively ascribed to the extremely compact structure evidenced by SEM images (see Figure 3c,d) which seems to jeopardize the interaction between the redox species (especially I_2_) and the hosting matrix. Albeit an active role of residual acetylcysteine or acetic acid (evidenced by FT-IR analyses, see Figure 2a) could not be completely ruled out, it seems to be just marginal in this case. Aiming at solving this issue, different tests have been performed varying the amount of the redox mediator added into the gel, but with only marginal improvements: as a matter of fact, if a lower amount of redox couple is added, the I_2_ evaporation is even faster; on the other hand, a higher amount of redox couple induces a partial phase separation even under mild stirring. Notwithstanding this, we decided to test the gel as the electrolyte: indeed, some examples in the literature report the use of sulfur-based additives able to interact with I_2_, inducing the modification of the electron distribution of the latter and causing a blueshift of its absorption, resulting in a more transparent electrolyte, so the discoloration of the gels could not be taken as a definitive proof of the unsuitability of the electrolyte.^59^ However, once implemented in complete aqueous DSSCs (see data for **CAC-I** and **CHA-I**), no photovoltaic efficiency could be recorded (PCE <0.05%) because of the almost null current density (*J*_SC_ <0.1 mA cm^–2^, Figure 4a). Also, Electrochemical Impedance Spectroscopy (EIS) measurements further support this hypothesis, showing a very resistive behavior in the **CAC-I** spectrum (R >150 KOhm, inset in Figure SI4), as expected by devices in which any current is flowing through. This result proves that both the hydrogels are likely unable to properly interact with I_2_ which tends to evaporate fast and completely from the gels during the mixing step, making them unsuitable for the proposed application.

**Figure 4:**
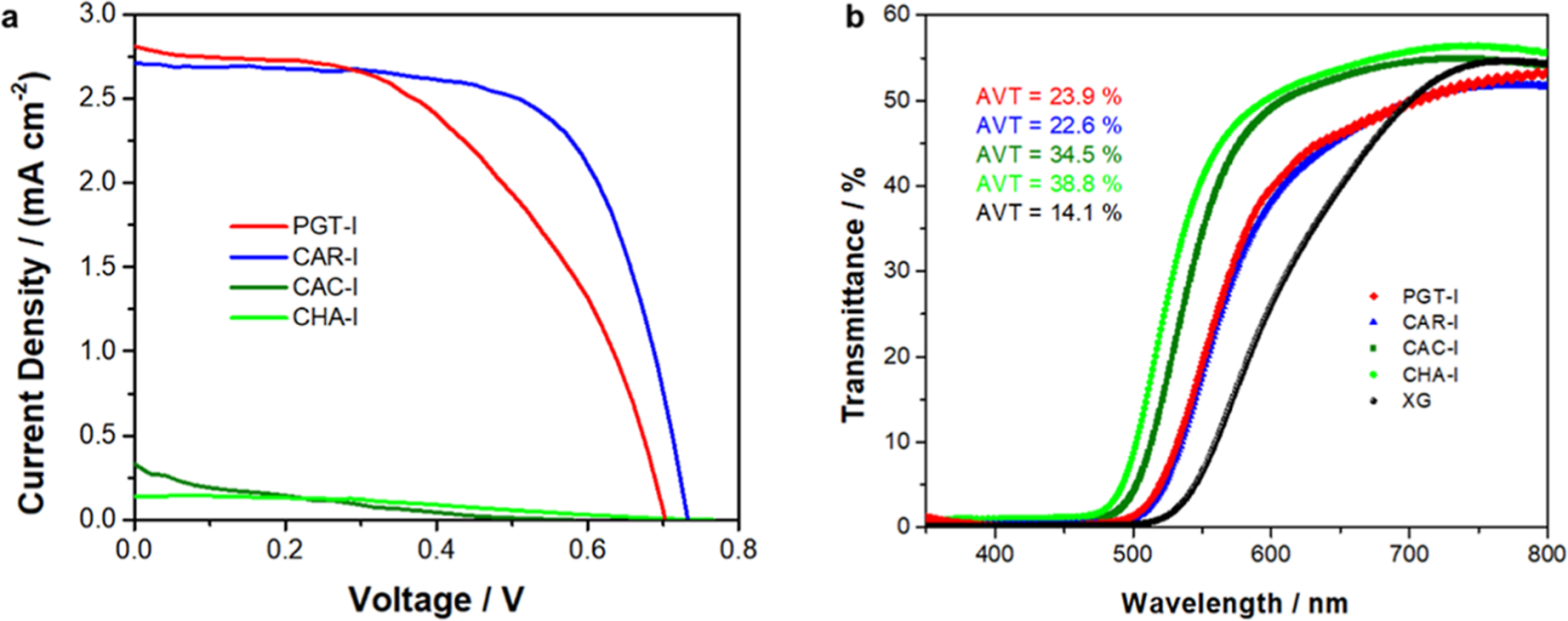
J–V curves (a) and UV–vis absorption spectra (b) of the most performing devices: **PGT-I** in red, **CAR-I** in blue, **CAC-I** in dark green, **CHA-I** in light green, and **XG** in black. In the inset of frame b, the AVT values have been listed, using the same color code.

After excluding **CAC-I** and **CHA-I** from further investigation, we focused our effort on the implementation of **CAR-I** and **PGT-I** as electrolytes for aqueous quasi-solid DSSCs. Both the hydrogels lead to decent photovoltaic performance (Figure SI5) mainly limited by the current density values which approach 3 mA cm^–2^ (Figure 4a), quite lower than the state-of-the-art value reached with the Xanthan Gum-based reference (see Table 2, entry 1). On the other hand, they prove themselves to be able to ensure a photovoltage approaching (**PGT-I**) or even overcoming (**CAR-I**) 700 mV, i.e., much higher than the one of the reference device (550 mV), as already highlighted in our recent publication dealing with similar systems using galactomannan as a capping agent in place of carrageenan or pork gelatin.^11^ Overall, **CAR-I** provides a photovoltaic conversion efficiency as high as 1.31% outperforming its counterpart based on gelatin (**PGT-I** PCE = 1%) mainly due to a higher Fill Factor (66% vs 52%). Notwithstanding this, the performances of **CAR-I** are still lower compared to that of the reference devices (PCE = 1.88%). A higher *V*_OC_ and a lower current density (resulting in a slightly lower efficiency) could be related to the different amounts of NaI present in the new gels. One should note that the xanthan-based reference electrolyte contains a higher concentration of Iodide (i.e., 5M). Indeed, alkali cations (e.g., Li^+^, Na^+^...) are known to cause a downshifting of the Fermi level of the TiO_2_ valence band, thus leading to a more effective electron injection from the dye’s LUMO to the TiO_2_ VB. This effect usually increases the current density values at the expense of the *V*_OC_ as a narrowing between the TiO_2_ VB and the redox potential of the mediator occurs.^19^ However, when the concentration in the xanthan-based electrolyte is reduced to 0.5M, the device efficiency scales down, as a result of the lower current density, leading to lower values compared to both **CAR-I** and **PGT-I**, further proving the promising behavior of the new quasi-solid aqueous electrolytes. This effect could be tentatively ascribed to a combination of different factors: looking at the electrode/electrolyte interface, the new biopolymer-based gels are expected to differently interact (and possibly passivate) the photoanode surface. Such a passivation could partially prevent alkali (from the redox mediator) or hydrogen (from water) ions from interacting (i.e., intercalating) into the titania layer, thus minimizing the downshift of the Fermi Level of the semiconductor. On the other hand, the presence of ZnO nanoparticles within the electrolyte could ensure a more stabilizing coordination of the mediator species (i.e., I^–^ and, at a lower extent, I_3_^–^) induced by both the presence of ZnO nanoparticles and the new biopolymers which may result in a downshift of the mediator’s redox potential (i.e., a deeper redox potential) and a consequent *V*_OC_ increase. A similar outcome has been noticed also in the case of the previously mentioned ZnO–galactomannan gel electrolytes.^11^ Supporting this hypothesis, some of us have recently reported on the unprecedented stabilization of I_2_ within a Deep Eutectic Solvent (DES) based on glycerol and choline Iodine, induced by the interaction of Iodine with the partially negatively charged hydroxyl oxygens involved in the hydrogen bond.^60^ Unfortunately, the quasi-solid nature and high concentration of the electrolyte prevent us from recording reliable cyclovoltammetric measurements to further corroborate our hypothesis. On the other hand, computational investigations are currently underway to rule out any possible impact of the chemical nature of the biopolymers on the position of the titania’s Fermi level. Notwithstanding this, the overall picture is further supported by EIS measurements (Figure SI4): both **CAR-I** and **PGT-I**’s EIS spectra show conventional shapes. Please note that, due to the lack of a proper equivalent circuit to fit quasi-solid aqueous DSSCs, we decided to keep the discussion on a qualitative scale, which gives fruitful insights though.^61^ At a closer inspection, **PGT-I** spectrum is characterized by a slightly larger first semicircle accounting for a higher charge transfer resistance at the counter electrode/electrolyte interface, as somehow expected by the lower fill factor, which, however, does not heavily impact the device behavior. The photovoltaic and electrochemical results match well with the morphological data reported in Figure 3a,b and Figure 3e for **CAR** and **PGT**, respectively, in which a channel-like structure could be evidenced, allowing the redox mediator to effectively flow throughout the gel.

**Table 2:** Summary of the Photovoltaic Figures of Merit Delivered by the Devices Discussed throughout the Paper. The Statistical Analyses Refer to the Average of 5 Nominally Identical Devices, at Least

	VOC/mV	JSC/(mA*cm^–2^)	fill factor/%	PCE/%
**XG-I-5** ^a^	552 ± 23	5.40 ± 0.12	62.8 ± 0.6	1.88 ± 0.03
**XG-I-0.5** ^b^	619 ± 54	2.52 ± 0.18	56.4 ± 2.6	0.88 ± 0.06
**CAC-I**	746 ± 34	0.23 ± 0.12	40.1 ± 7.0	0.07 ± 0.03
**CHA-I**	561 ± 23	0.12 ± 0.08	30.6 ± 5.2	0.02 ± 0.02
**CAR-I**	723 ± 4	2.66 ± 0.03	66.3 ± 0.7	1.28 ± 0.05
**PGT-I**	701 ± 3	2.80 ± 0.01	49.7 ± 0.7	0.98 ± 0.01
**PGT-CS-I**	658 ± 13	2.50 ± 0.16	73.5 ± 0.7	1.20 ± 0.06
**PGT-CW-I**	710 ± 8	3.27 ± 0.05	67.3 ± 0.2	1.56 ± 0.04
**PGT-CA-I**	664 ± 25	2.52 ± 0.03	72.9 ± 0.6	1.22 ± 0.05
**CAR-CS-I**	629 ± 10	1.62 ± 0.41	54.4 ± 14.8	0.54 ± 0.02

a Electrolyte composition: NaI = 5 M and I_2_ = 0.03 M in a CDCA-saturated aqueous solution jellified with 3% w/w of xanthan gum.

b Electrolyte composition: NaI = 0.5 M and I_2_ = 0.03 M in a CDCA-saturated aqueous solution jellified with 3% w/w of xanthan gum.

The tunable semitransparency of DSSCs is an added value compared to conventional photovoltaic devices. Therefore, besides photoconversion efficiency, we also recorded some UV–vis spectra of the most efficient devices (see Figure 4b), aiming at evaluating their AVT (Average Visible Transmittance) values.^62^ The highest values were obtained for **CAC-I** and **CHA-I** devices, as widely expected by the absence of iodine within the gels. On the other hand, a slightly higher AVT value is measured for **PGT-I** (23.9%) with respect to **CAR-I** counterparts (22.6%). More interestingly, both clearly outperform the Xanthan-gum-based reference device (AVT = 14.1%). To monitor the best trade-off between transparency and efficiency of semitransparent photovoltaic devices, a new figure of merit has been recently introduced, namely, the Light Utilization Efficiency (LUE) measured as the product between the AVT and the PCE of a single device. In our case, **CAR-I** showed an improved LUE compared to both **PGT-I** (+6%) and the reference device based on xanthan gum (+70%, **XG**). It should be noted that both the counter electrode and the thickness of TiO_2_ were not specifically optimized for their applications in semitransparent devices. Similarly, also the dye (i.e., D131, which absorbs light up to 500 nm^63^) has been selected aiming at maximizing the photovoltaic conversion efficiency in conjunction with a quasi-solid aqueous electrolyte more than the device transparency.

Besides the different concentration of NaI, the higher photovoltaic efficiency of the reference electrolyte using xanthan gum as the gelling agent could in part be related to the use of a saturated CDCA aqueous solution as the mobile phase of the hydrogel.^12^ Indeed, it has been proved that the latter can play an important role as a passivating agent on top of the dye–TiO_2_ surface, minimizing the recombination reaction at the photoanode/electrolyte interface.^64^ In a recent work,^11^ we purposely avoid the use of CDCA, which would partially jeopardize the sustainability of the electrolyte: as a matter of fact, the implementation of the right amount of CDCA into the electrolyte is a quite energy and material demanding process (requiring a day-long stirring process at 50 °C to ensure its solubilization in water). Here, to shine light on the interaction of CDCA with our new gels, we decided to implement it in the most performing systems, namely, the ones based on pork gelatin and carrageenan. However, the synthetic procedure (i.e., the in situ formation of Zn-based nanoparticles which is highly pH dependent^65^) prevents us from using the same approach employed in the formation of the XG-based reference gel. Following from that, we decided to introduce CDCA only after the formation of the nanoparticles and prior to the first centrifugation step (see the experimental part for further details). One should note that the proposed approach, while ensuring the presence of CDCA within the electrolyte, does not allow a quantitative control of the additive concentration within the gel, but the latter is expected to be quite lower compared to the one of the **XG-I** reference electrolytes, as also evidenced by the absence of specific peaks in the FT-IR spectra (Figure 2b–d). Following from that, the presence of CDCA seems not to impact the I-based redox mediator loading capability of the resulting hydrogels. More in detail, the presence of CDCA seems not to impact the I-based redox mediator loading capability of the resulting hydrogels, likely due to the relatively low amount of CDCA dissolved as also evidenced by the absence of specific peaks in the FT-IR spectra (Figure 2b–d). More in detail, the presence of CDCA within the electrolyte solution seems to have a different effect if pork gelatin and carrageenan are employed as gelling agents. On the one hand, CDCA seems to mitigate the low FF (50%) measured for **PGT-I**, which reaches the impressive value of 73.5% for **PGT-CS-I**, even if with slightly lower *J*_SC_ (2.50 mA*cm^–2^ vs 2.80 mA*cm^–2^) and *V*_OC_ (660 mV vs 700 mV): the overall effect is a 20% increase of the photovoltaic efficiency (see Table 2 and Figure 5). On the other hand, a negative influence of CDCA in **CAR-CS-I** is clear from the sharp downgrading of all the photovoltaic figures of merit compared to the CDCA-free counterparts (e.g., PCE = 0.54% vs 1.31%, respectively). The opposite behavior of the two electrolytes could be tentatively rationalized looking at the different chemical functionalities exposed by the biopolymers: indeed, the CDCA, being a weak acid in water, can induce the partial protonation of the amine and amide moieties of **PGT**, impacting on the hydrogen-bond network within the electrolyte. The newly established network could free the redox mediator movement improving the charge diffusion and, consequently, the fill factor. As a counterpart of this, the lower coordination causes the redox mediator to have a lower redox potential leading to a decrease in *V*_OC_, which also the slightly lower *J*_SC_ could be related to. Even if the redox mediator could induce some modifications in the hydrogen-bond network within the gel, the higher mobility of the redox mediator seems to be somehow counterintuitive considering the morphology of **PGT-CS** evidenced by SEM images (Figure 3g); the latter shows a more compact structure theoretically leading to hindering of charge diffusion. However, one should consider that the freeze-drying process required to collect SEM images of the gels would induce a partial collapse of the gels’ structure. Following on from this, the presence of charged moieties is expected to give rise to the more compact structure evidenced. On the other hand, the absence of prone-to-protonation moieties in **CAR** will prevent a fruitful chemical interaction between the biopolymer and the additive. This results in a more dense electrolyte, which could form a passivation layer on the electrodes, minimizing the current density provided by the device and limiting the FF as well. Also, *V*_OC_ is lower compared to the CDCA-free counterpart. This could be tentatively rationalized as follows: the acid moieties of the additive, if not used in forming hydrogen bonds, could interact with I^–^, partially limiting its interaction with Na^+^ and preventing the formation of relatively strong Na^+^–I^–^ ionic couples. This would cause a higher concentration of free alkali cations, which can accumulate at the photoanode/electrolyte interface, downshifting the TiO_2_ Fermi Level. Overall, whereas the addition of CDCA seems to be positive for amine-/amide-containing biopolymers, it is highly detrimental for carboxylate-free sugars, opening the way for the implementation of coadditive different from CDCA.

**Figure 5:**
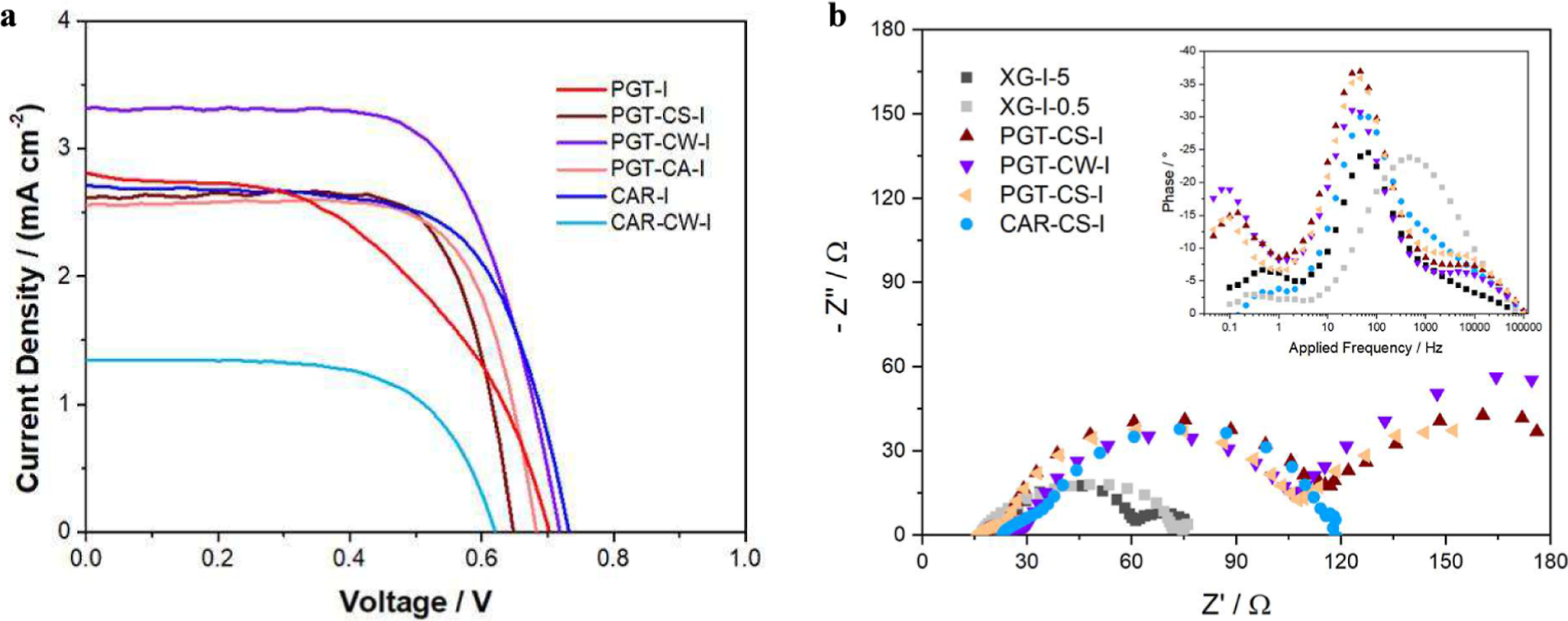
J–V curves (a) of the most performing devices based on CDCA-modified hydrogel electrolytes: **PGT-I** in red, **PGT-CS-I** in wine red, **PGT-CW-I** in violet, **PGT-CA-I** in pink, **CAR-I** in blue, and **CAR-CS-I** in light blue; (b) Nyquist and Bode (inset) plots of the most performing CDCA-containing electrolytes: **XG-I-5** in black, **XG-I-0.5** in gray, **PGT-CS-I** in wine red, **PGT-CW-I** in violet, **PGT-CA-I** in pink, and **CAR-CS-I** in light blue.

Based on these preliminary results, we decided to further tune the implementation of CDCA by slightly changing the incorporation procedure (see the Experimental Section). The washing of the CDCA-containing gel with a CDCA-saturated aqueous solution (**PGT-CA-I**) does not lead to any improvement compared to the original electrolyte (**PGT-CS-I**) suggesting that the maximum amount of CDCA is already embedded in the gel during the main centrifugation step, as also evidenced by morphological analyses. On the other hand, the washing of CDCA-free gel with a CDCA-saturated aqueous solution (**PGT-CW-I**) leads to a clear improvement in the photovoltaic conversion efficiency (1.56%, +25% compared to **PGT-CS-I**), mainly related to a sharp increase in the current density value which approaches 3.5 mA*cm^–2^ (see Figure 5a). Once more, the morphology of the gels and the establishment of a wider and more functional hydrogen-bond network seems to play a crucial role being the texture of the latter denser when the CDCA is added right before the first centrifugation step. On the other hand, the use of a saturated CDCA solution only during the washing step likely leads to just a marginal incorporation of the additive within the electrolyte, thus only slightly impacting the chemistry and the morphology of the gels. This brings us to postulate that a controlled implementation of CDCA within the hydrogel electrolyte based on amine- and amide-containing biopolymers is the most promising strategy to further improve the photoelectrochemical performances of our electrolytes. Further studies are currently ongoing to evaluate the actual sustainability impact of the CDCA-modified electrolytes and to ensure that the latter is positively counterbalanced by the efficiency improvement.

However, some preliminary EIS spectra of the most performing CDCA-containing electrolytes have been recorded. As previously stated, due to the absence of a proper equivalent circuit to interpolate experimental data,^61^ we decide to keep the discussion on a semiquantitative plane to avoid any overinterpretation and/or speculation on the data. As already reported in Figure SI4, carrageenan-derived electrolytes lead to a more compact shape of the EIS spectrum with the absence of an overlapping between the second (related to photoanode/electrolyte charge transfer) and the third (related to diffusion throughout the electrolyte) semicircle. On the other hand, EIS spectra of porcine gel-derived electrolytes present a clear separation between the second and third semicircle; this is representative for a much higher resistance to redox mediator diffusion which seems not to meaningfully impact the photovoltaic properties of the final devices though. One should note that reference devices (i.e., **XG-I-5** and **XG-I-0.5**) both show much smaller EIS spectra, usually indicating a less resistive behavior. However, this could not be (without a proper data interpolation by using a Transmission-Line model^66^) used as a litmus test of the photoelectrochemical behavior of the device, as the behavior could be alternatively ruled by an extremely fast charge transport and a slower charge recombination (higher efficiency device) or by an extremely fast charge transport and recombination (lower efficiency device). In order to get more information from EIS, we looked at Bode’s plot (applied frequency vs impedance phase, inset in Figure 5b). Here, a clear difference could be seen between **XG-I-5** (black dots) and **XG-I-0.5** (gray dots), with the latter showing the maximum of the first peak at much higher applied frequency values (474 Hz, corresponding to a characteristic time of 2 ms). This implies a much faster (and unwanted) charge recombination, giving rise to the lower photovoltaic performance. With respect to the phase behavior of natural polymer-derived electrolytes, one can note how the position of the main peak is very similar to the one of **XG-I-5** with characteristic time values slowing down in the order **XG-I-5** (64 Hz, 16 ms), **CAR-CS-I** (54 Hz, 19 ms), **PGT-CA-I** ≈ **PGT-CW-I** ≈ **PGT-CS-I** (37 Hz, 27 ms).

The slowing down of the charge recombination time found for the PGT-derived systems leads to well-performing devices; however, the performance is still lower compared to that of **XG-I-5**; this could be tentatively ascribed to a concurrent slowing down of the charge transport kinetics (whose behavior could not be extracted from Bode’s plot). Finally, the second peak for all the PGT-derived devices is located at a much lower applied frequency (≈0.1 Hz, 10s), supporting what has been evidenced by the Nyquist plot description.

Dye-Sensitized Solar Cells have been recently proposed as one of the most promising indoor photovoltaic technologies with PCE values surpassing 30%.^67,68^ However, the state-of-the-art indoor DSSCs are conventionally assembled with organic copper-based electrolytes, whereas only few examples report on aqueous DSSCs.^69,70^ To further expand the applicability of our biopolymer-derived electrolyte, we tested them under indoor lightning (i.e., 1200 lx). In order to ensure a reliable comparison with the **XG**-based reference electrolyte, only CDCA-containing gels are tested. As one can see from Figure 6a and Table SI1, for current density values, the same trend as under 1 Sun is obtained, with the new biopolymer-based electrolytes behaving similarly to **XG-I-5**. The lower light intensity also reflects on the lower current density values, which do not exceed 0.04 mA cm^–2^ and which lead to FF values constantly higher than 70%. Additionally, all of the **PGT**-based electrolytes show extremely similar figures of merit, highlighting that the way in which the CDCA is added to the electrolyte plays a crucial role only when a higher current is provided. They also outperform **CAR-CS-I**, although the difference is smaller compared to the outdoor light measurement. Reducing the amount of light shining on the device, a downshifting of the TiO_2_ VB is expected, as proved by the lower *V*_OC_ generated by the device (ranging from 0.38 V for **XG-I-5** and ca. 0.58 V for **PGT**-based electrolytes); notwithstanding this all the biopolymer-based electrolytes ensure a much higher *V*_OC_. Overall, no matter the lower absolute values of the single figures of merit, all of the devices showed higher performance when operating under indoor illumination (see Table SI1), with efficiency around 3.0%. Very interestingly, under low-light intensity, **PGT**-based electrolytes outperform the reference **XG-I-5** of about 25% (3.18% vs 2.40% for **PGT-CS-I** and **XG-I-5**, respectively), opening the door for further investigation of these electrolytes for next-generation indoor aqueous DSSCs.

**Figure 6:**
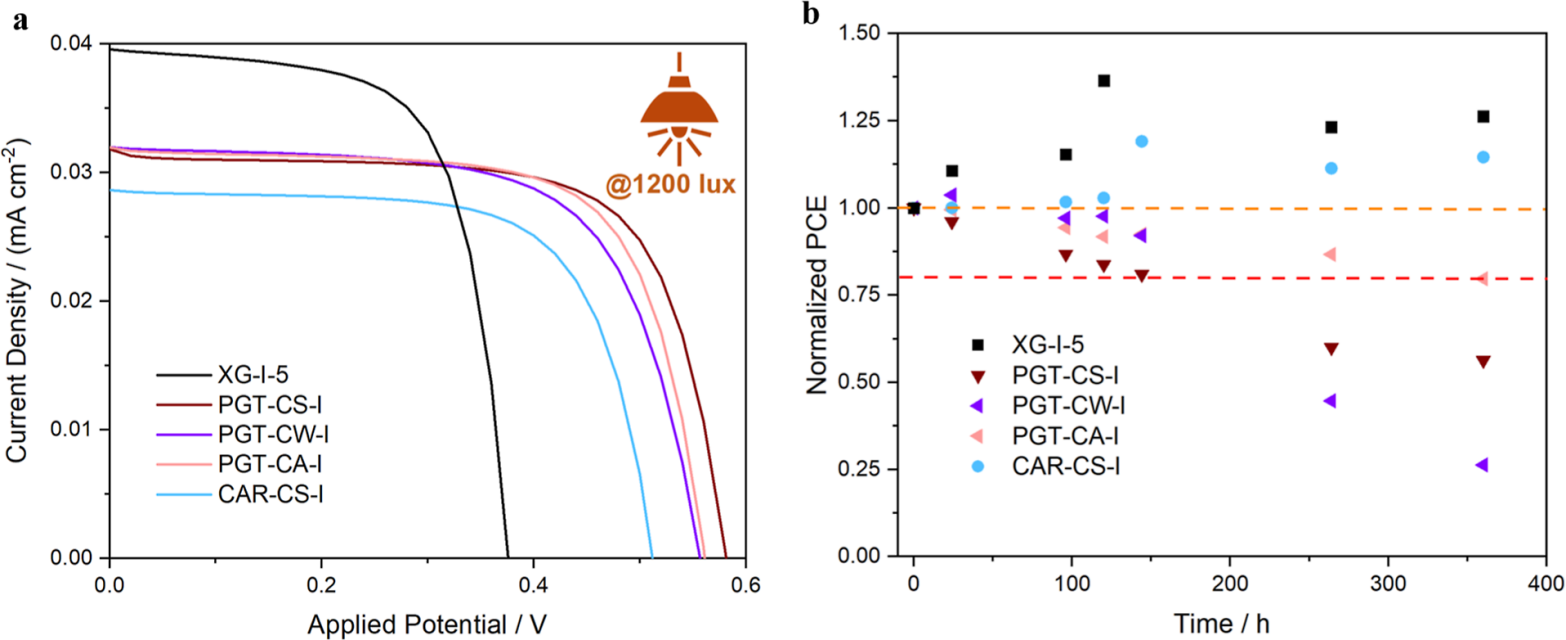
Indoor light (@1200 lx) J–V curves (a) and 1 Sun PCE stability trends over time (b) of the most performing CDCA-containing electrolytes: **XG-I-5** in black, **PGT-CS-I** in wine red, **PGT-CW-I** in violet, **PGT-CA-I** in pink, and **CAR-CS-I** in light blue.

As a conclusive analysis, we tested the stability of our devices under shelf life conditions. Also, in this case, for a more reliable comparison with XG-based electrolytes, only CDCA-containing electrolytes were screened. As shown in Figure 6b, the devices could be clustered in two groups: from the one hand, **XG**-based and **CAR**-based devices experiment an increase in the normalized PCE values in the first 120 h (5 days) of aging, whereas the **PGT**-based ones do not show any PCE increase. One should note that the trend in PCE mirrors that of *J*_SC_ (Figure SI6), while both FF and *V*_OC_ exhibt only minor deviations. This behavior could be once more related to the difference in the chemical nature of the biopolymers, with the ones based on pork gelatin (and rich in amine/amide moieties) showing a more open morphology in the wet state, allowing a very fast permeation of the redox mediator; on the contrary, the slower I-species diffusion in linear sugar-based gels is likely due to a more compact morphology, accounting for the longer PCE stabilization period. Very interestingly, the clustering of the device is also preserved on longer periods, with the **PGT**-based electrolytes showing a clear PCE drop after less than 1 week (*t* <168 h); among them, only **PGT-CA-I** is able to reach a T80 (i.e., the time at which the PCE drops down to 80% of its initial value) longer than 360 h. Very interestingly, no matter the lower initial PCE (Figure 5 and Table 2), **CAR-CS-I** emulates **XG**-based devices, showing normalized PCE values higher than 100% even after 15 days of storage under shelf life conditions. The overall picture which emerges from these analyses tells about a not-straightforward interaction of CDCA with the different biopolymer-based electrolytes. On the one hand, it allows one to increase both the FF and the PCE of the **PGT**-based device, but it does not ensure any long-term stabilization of the latter. On the other hand, CDCA has been proved to be extremely detrimental for the performances of **CAR**-based devices, but it seems to play a role in prolonging their lifetime.

## Conclusions

Throughout this paper, different hydrogels derived from Zn-based nanoparticles and biopolymers are designed and formulated by exploiting a truly green synthetic procedure. The gels are thoroughly characterized from a chemical, structural, and morphological point of view, by means of FT-IR, XRD, and SEM imaging, respectively, pinpointing their implementation as the electrolyte for quasi-solid aqueous Dye-Sensitized Solar Cells. The chemical nature of the biopolymer substantially influences the properties of hydrogel and its ability to host a redox couple based on NaI and I_2_: for example, the use of chitosan as a polymeric scaffold results in an ineffective stabilization of iodine who tends to evaporate fast; on the other hand, both carrageenan and pork gelatin in conjunction with ZnO nanoparticles are proved to lead to quasi-solid electrolytes which approach or even overcome a photovoltaic efficiency of 1% (i.e., PCE = 0.98% or 1.28% for pork gelatin- and carrageenan-based gels, respectively), exhibiting an impressively high open-circuit potential (up to 700 mV). Relying on these very promising results, the hydrogels were further engineered by incorporating CDCA which was implemented during the synthesis for the first time. Quite interestingly, the effect of CDCA addition, which is detrimental for carrageenan-based electrolytes (PCE = 0.54%, more than halved), induces a clear boost in the photovoltaic performance of porcine gelatin electrolytes (PCE = 1.20%), as expected by the different chemical nature of the biopolymers. A more controlled implementation of CDCA, whose amount within the electrolyte is minimized by adding it only during the final washing procedure of the gel, allows further increase of the performance of the resulting device which overcomes a PCE as high as 1.5%. As an added value, the newly designed electrolytes show an improved light transmittance in the visible range, allowing the fabrication of semitransparent quasi-solid aqueous DSSCs which has a 55% higher Average Visible Transmittance and a 20% better Light Utilization Efficiency. More importantly, the devices based on CDCA-engineered porcine gel-derived electrolytes provide a remarkable photoconversion efficiency under indoor lighting (i.e., 1200 lx) approaching 3% and outperforming the reference device based on xanthan gum. On the other hand, carrageenan-based counterparts, properly modified with CDCA, counterbalanced a relatively low photoconversion efficiency with a remarkably long-term stability: indeed, they did not lose any efficiency after 400 h of shelf life aging, emulating the behavior of the reference devices. In summary, our work paves the way toward the exploitation of innovative and green but effective hydrogel electrolytes for the next generation of aqueous Dye-Sensitized Solar Cells coupling good photovoltaic performance with improved aesthetic features and better sustainability.

## Supplementary Material



## Notes

Chemical structure of D131 dye; zoomed SEM images of sample CAR and SEM image of sample PGT-CA; photographs of iodine-loaded gels of samples CAC-I, CAR-I, PGT-I, and XG and the resulting DSSCs studied in this work; electrochemical impedance spectra of sample PGT-I, CAR-I, CAC-I, and CHA-I; PCE, JSC, VOC, and FF (box plots) of sample XG-I-5, PGT-I, CAR-I, PGT-CW-I, PGT-CS-I, PGT-CA-I, and CAR-CS-I; VOC, JSC, and FF stability trends over time of sample XG-I-5, PGT-CS-I, PGT-CW-I, PGT-CA-I, and CAR-CS-I; and summary of the photovoltaic figures of merit of sample XG-I-5, PGT-CS-I, PGT-CW-I, PGT-CA-I, and CAR-CS-I (PDF)
