# Supplementary material for: Strategies for the Implementation of Chenodeoxycholic Acid in Natural Polymer-Based Hydrogels for Stable and Low-Light Effective Quasi-Solid Electrolytes for Aqueous Dye-Sensitized Solar Cells
Source: ACS Appl Energy Mater. 2025 Nov 25;8(23):17451–66. doi: 10.1021/acsaem.5c02940 (PMC12691169; doi:10.1021/acsaem.5c02940)
Supplement: Supplementary file 1 [file ae5c02940_si_001.pdf]

Supporting Information

Strategies for the implementation of Chenodeoxycholic acid in natural polymers-based hydrogels for stable and low-light effective quasi-solid electrolytes for aqueous Dye-Sensitized Solar Cells

Lorenzo Casoli[1#], Ana Yancy Segura Zarate[2,3#], Elvira Maria Bauer[4*], Matteo Bonomo[2], Simone Galliano[2], Claudia Barolo[2,5,6*], Angelo Lembo[1], Lorenzo Gontrani[1], Marilena Carbone[1,4*]

[1] STARTNETICS - Department of Chemical Science and Technologies, University of Rome Tor Vergata, Via della Ricerca Scientifica 1, 00133 Rome, Italy

[2] Department of Chemistry, NIS Interdepartmental Center and INSTM Reference Centre, University of Torino, Via Gioacchino Quarello 15/a, 10135 Torino, Italy

[3] Escuela de Fisica, Instituto Tecnologico de Costa Rica, Cartago 159-7050, Costa Rica

[4] Institute of Structure of Matter - Italian National Research Council (ISM-CNR), c/o Area della Ricerca di Roma1, Strada Provinciale 35d n. 9, 00010 Montelibretti, Italy

[5] Italian National Research Council - Institute of Science, Technology and Sustainability for Ceramics (ISSMC-CNR), Via Granarolo 64, 48018 Faenza, RA, Italy

[6] ICxT Interdepartmental Center, University of Turin, Lungo Dora Siena 100, 10153 Torino, Italy

[#]these authors equally contributed to the paper

[*]corresponding authors: Elvira.Bauer@ism.cnr.it, Claudia.Barolo@unito.it; carbone@uniroma2.it

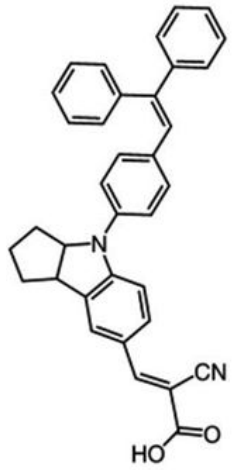

Figure SI1. Chemical structure of dye D131.

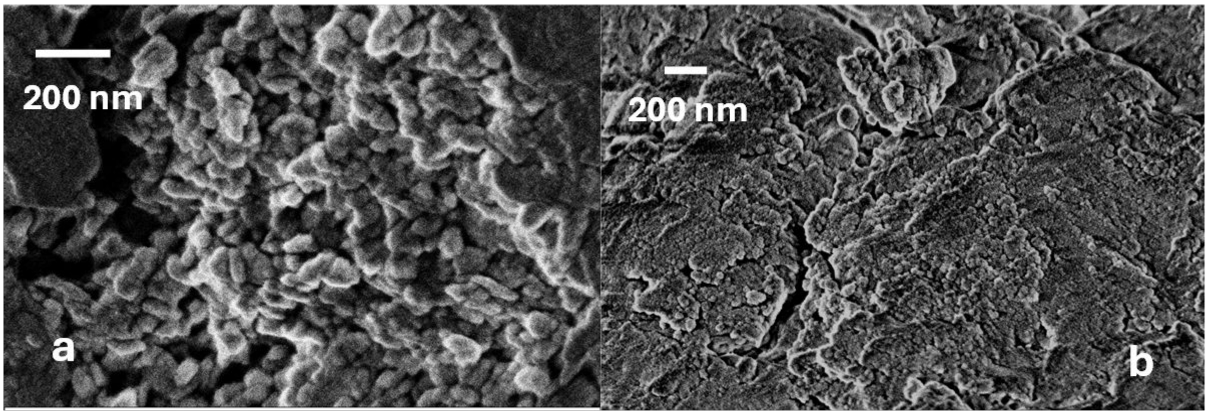

Figure SI2. a) Zoomed SEM images of Figure 3a (200K X) and b) SEM image of PGT-CA sample.

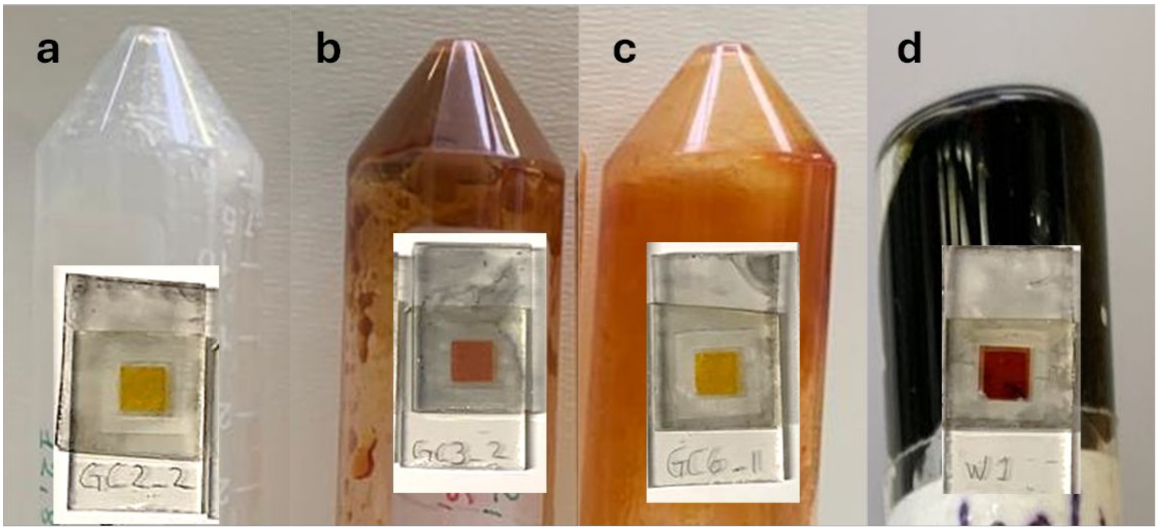

Figure SI3. Digital photographs of the iodine-loaded gels and the resulting DSSCs studied throughout the paper: a) **CAC-I**, b) **CAR-I**, c) **PGT-I** and) **XG**. Please note that the yellowish colour of **CAC-I** is due to the D131 dye only.

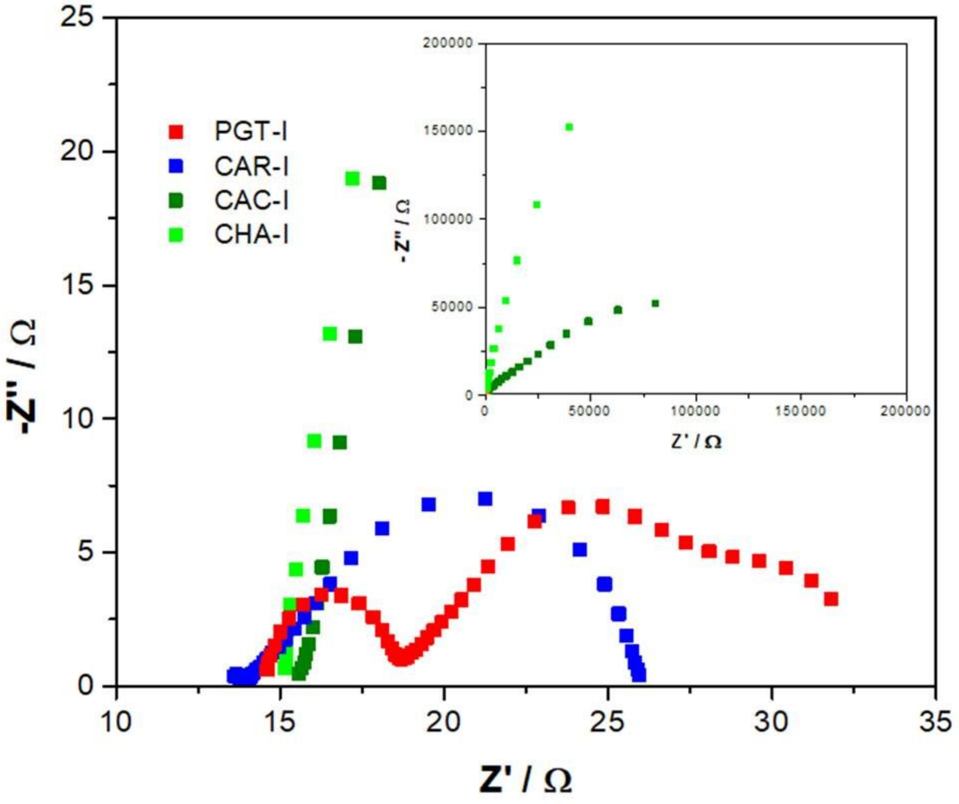

Figure SI4. Electrochemical Impedance Spectra (shown as Nyquist's plot) of **PGT-I** (red squares), **CAR-I** (blue squares), **CAC-I** (dark green squares) and **CHA-I** (light green squares) based devices. The inset shows the full-scale spectra of **CAC-I** and **CHA-I**.

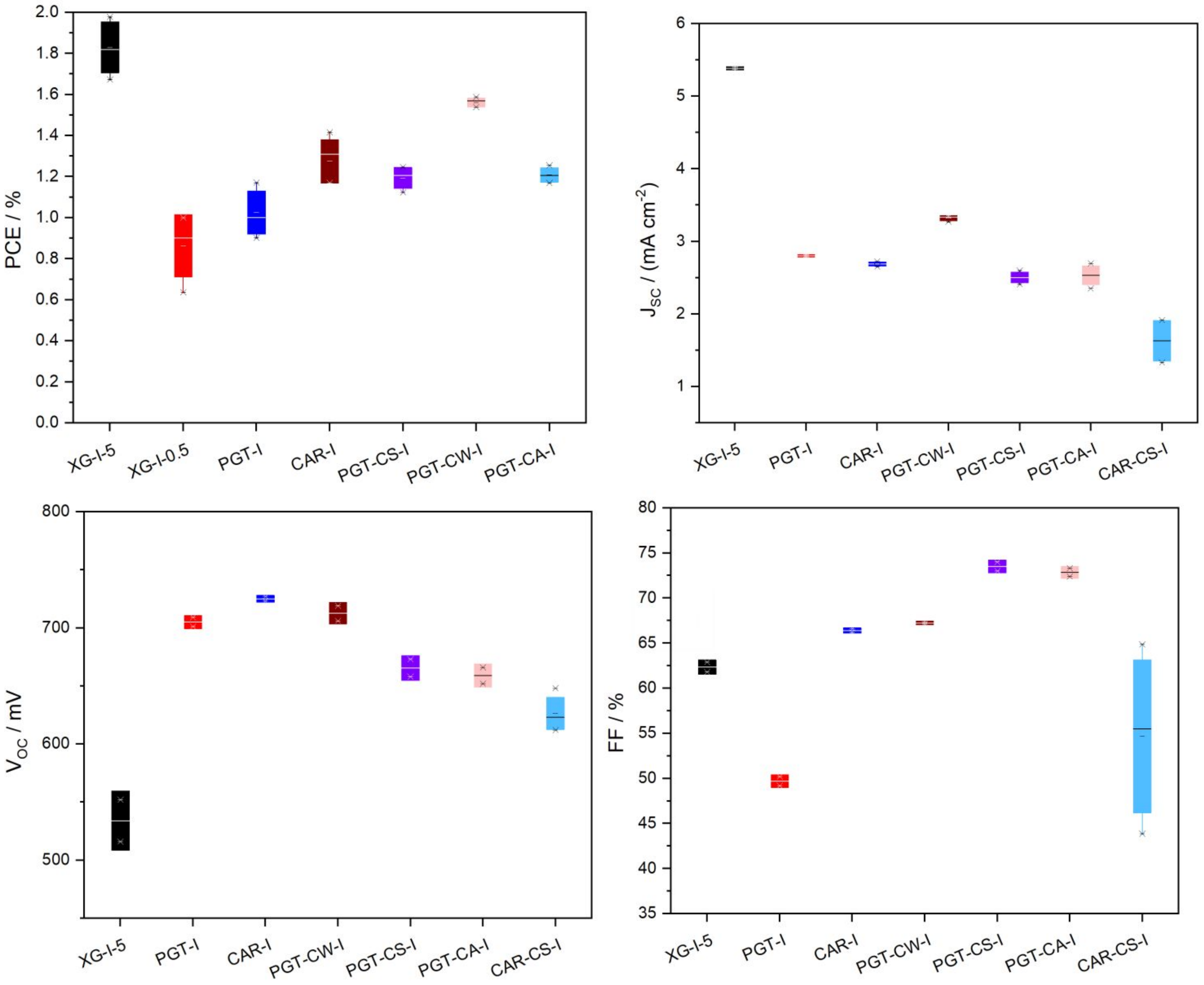

Figure SI5. Box-plot of PCE (top left), JSC (top right), VOC (bottom left) and FF (bottom right) of the devices reported in Table 2: **XG-I-5** in black, **PGT-I** in red, **CAR-I** in blue, **PGT-CW-I** in wine-red, **PGT-CS-I** in violet, **PGT-CA-I** in pink and **CAR-CS-I** in light blue.

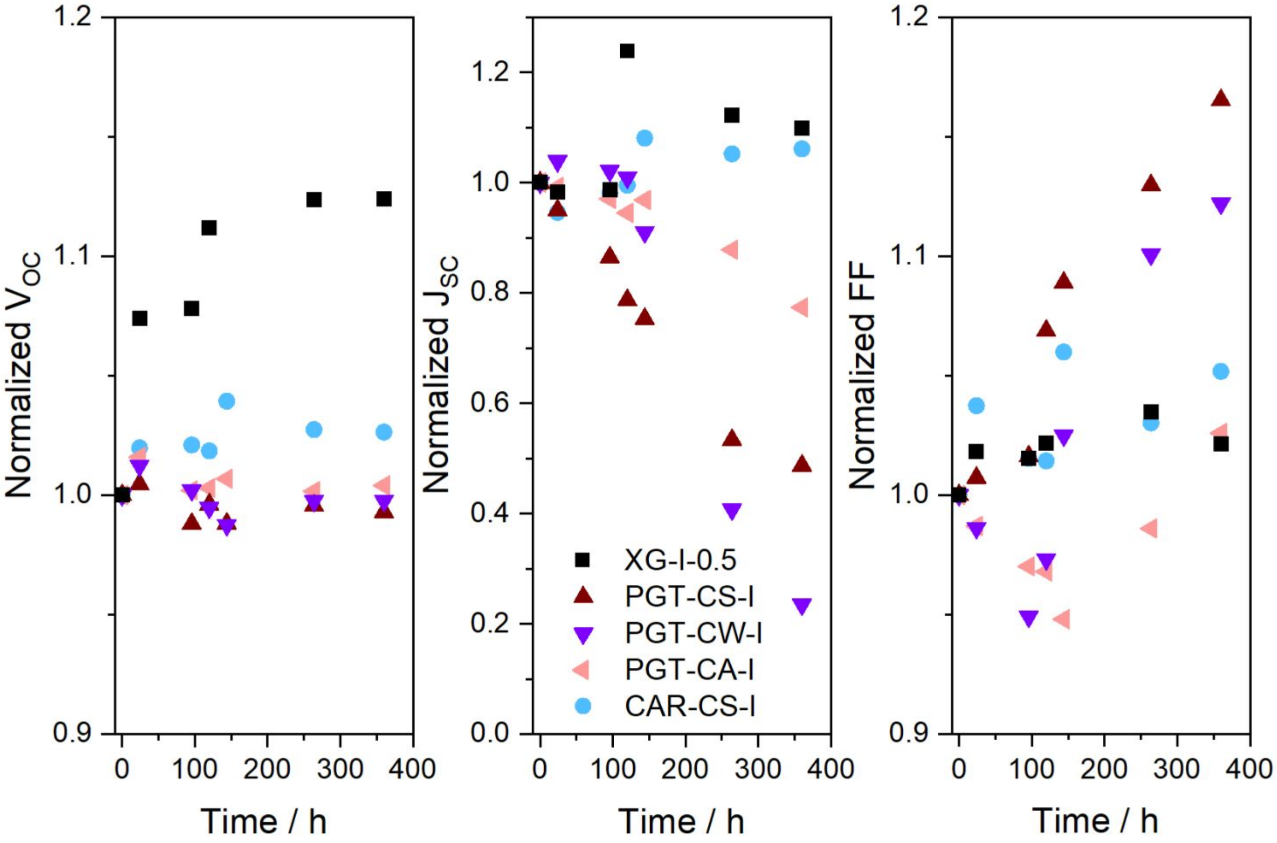

Figure SI6. VOC (left), JSC (middle) and FF (right) stability trends over time of the most performing CDCA-containing electrolytes: **XG-I-5** as black squares, **PGT-CS-I** as wine-red triangles, **PGT-CW-I** as violet triangles, **PGT-CA-I** as pink triangles and **CAR-CS-I** as light blue circles.

Table SI1. Summary of the photovoltaic figures of merit delivered by the most performing CDCA-containing electrolytes under indoor light at 1200 lux.

	V_{OC} / mV	J_{SC} / (mA*cm^{-2})	Fill Factor / %	PCE / %
**XG-I-5[a]**	376	0.0396	66.8	2.40
**PGT-CS-I**	584	0.0316	71.4	3.18
**PGT-CW-I**	560	0.0307	71.3	2.96
**PGT-CA-I**	562	0.0318	70.4	3.04
**CAR-CS-I**	513	0.0286	68.8	2.44

[a] Electrolyte composition: NaI = 5M and I_2 = 0.03M in a CDCA-saturated aqueous solution jellified with 3% w/w of xanthan-gum.
